# Early detection of unilateral ureteral obstruction by desorption electrospray ionization mass spectrometry

**DOI:** 10.1038/s41598-019-47396-x

**Published:** 2019-07-29

**Authors:** Shibdas Banerjee, Anny Chuu-Yun Wong, Xin Yan, Bo Wu, Hongjuan Zhao, Robert J. Tibshirani, Richard N. Zare, James D. Brooks

**Affiliations:** 1 Department of Chemistry, Stanford University, Stanford, CA 94305 USA; 2 Department of Chemistry, Indian Institute of Science Education and Research Tirupati, Tirupati, 517507 India; 3 Department of Urology, Stanford University School of Medicine, Stanford, CA 94305 USA; 4 Departments of Biomedical Data Sciences, and of Statistics, Stanford University, Stanford, CA 94305 USA

**Keywords:** Urinary tract obstruction, Diagnostic markers

## Abstract

Desorption electrospray ionization mass spectrometry (DESI-MS) is an emerging analytical tool for rapid *in situ* assessment of metabolomic profiles on tissue sections without tissue pretreatment or labeling. We applied DESI-MS to identify candidate metabolic biomarkers associated with kidney injury at the early stage. DESI-MS was performed on sections of kidneys from 80 mice over a time course following unilateral ureteral obstruction (UUO) and compared to sham controls. A predictive model of renal damage was constructed using the LASSO (least absolute shrinkage and selection operator) method. Levels of lipid and small metabolites were significantly altered and glycerophospholipids comprised a significant fraction of altered species. These changes correlate with altered expression of lipid metabolic genes, with most genes showing decreased expression. However, rapid upregulation of PG(22:6/22:6) level appeared to be a hitherto unknown feature of the metabolic shift observed in UUO. Using LASSO and SAM (significance analysis of microarrays), we identified a set of well-measured metabolites that accurately predicted UUO-induced renal damage that was detectable by 12 h after UUO, prior to apparent histological changes. Thus, DESI-MS could serve as a useful adjunct to histology in identifying renal damage and demonstrates early and broad changes in membrane associated lipids.

## Introduction

Renal biopsy is used commonly to assess the etiology of renal failure, where it has been reported to alter clinical management in approximately 40% of all patients and up to 70% of cases with acute kidney injury^1,2^. Between 2008 and 2012, 118,064 renal biopsies, excluding transplant patients and those under age 18, were recorded in hospital admissions from the Nationwide Inpatient Sample database, which captures approximately 20% of admissions in the U.S^3^. Assessment of renal biopsy relies on histopathology, although immunohistochemistry for protein markers for specific diseases or cell types can be employed to form a diagnosis. Discovery-based approaches, like transcriptomics, proteomics, and metabolomics, have been used to identify biomarkers of renal damage and have been proposed as a means to distinguish the cause of renal damage^4–8^. However, because of cost, the need for significant amounts of tissue that would exhaust a biopsy sample, and time-consuming sample preparation, these approaches do not lend themselves to use in a clinical setting.

Desorption electrospray ionization mass spectrometry (DESI-MS) is an emerging tool for rapid, label-free assessment of tissue metabolomic profiles and can be used for metabolome-based spatial imaging (DESI-MSI)^9,10^. DESI-MS operates under ambient conditions (room temperature and atmospheric pressure), requires minimal tissue preparation and can be performed rapidly, allowing for easy integration into the clinical work-flow. Unlike other MS-based tissue imaging technologies such as MALDI (matrix-assisted laser desorption/ionization), DESI-MS does not require a vacuum chamber or a special chemical matrix for ionization and causes little tissue destruction, thereby allowing conventional histological assessment of tissues after the MS study. DESI-MS is being tested for applications in oncology, where metabolic signatures have been identified that distinguish normal from malignant tissues from several sites including stomach, breast, pancreatic, prostate and brain cancers^11–16^. Because DESI-MS can be performed rapidly, it is being developed for the assessment of tumor margins and involvement of lymph nodes in almost real time to inform decision-making during surgical resections^14,15^. DESI-MS has also been used as a discovery platform to define spatial and temporal metabolomic changes in oncogene-induced cancer model systems^13,17–19^. In these studies, previously unknown metabolic changes, particularly in lipids that have potential roles in cell signaling, have been revealed and have possible therapeutic implications.

DESI-MS has seen more limited use outside of oncology^9,20^. Our group has used DESI-MS to analyze fingerprints, and, based on lipid signatures, can impute age, ethnicity, and gender with sufficient accuracy to suggest a role in forensic investigations^21^. To evaluate whether DESI-MS could provide information from renal tissues, such as those obtained from a renal biopsy, we tested a commonly used mouse model of renal damage, unilateral ureteral obstruction (UUO). This well-characterized model system allows assessment of metabolic changes over a time course. We were interested to determine whether metabolic changes could be observed in renal tissues after injury and whether they could be used to develop predictive models that identified renal compromise early.

## Results

### DESI-MS study on kidney tissue sections

**Figure 1:**
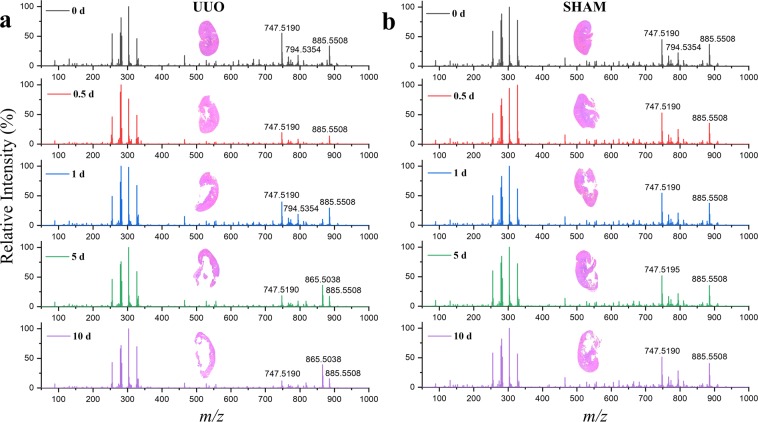
Temporal profiles of ion signals in UUO and sham control. Average DESI-mass spectra (normalized) in negative ion mode for kidney tissue specimens collected from (**a**) eight UUO and (**b**) corresponding eight sham-operated mice at each time point (0 d, 0.5 d, 1 d, 5 d, and 10 d). See Table S1 in SI for the identification of some important and abundant species with different *m/z* values. Inset of each panel shows the optical image (H&E) of a typical kidney specimen corresponding to the given time point.

To investigate the dynamic profile of metabolites and lipids in UUO by DESI-MS, we performed surgical UUO on the left kidney of 40 mice as described previously^8^. Kidneys were harvested at five time points: 0 d, 0.5 d, 1 d, 5 d, and 10 d (n = 8 per time point). In parallel, we harvested the left kidney of sham-operated mice at the same time points as controls. Sections of the fresh frozen kidney tissue samples (15-µm thickness) were analyzed rapidly by using DESI-MS. Figure 1 shows average DESI mass spectra in negative ion mode acquired after 1 min scribble scanning at each time point for the UUO (left panels) and sham control (right panels). In both cases, molecular species from the kidney tissue were detected in the 50–1000 *m/z* range. We identified and characterized several species from the data (Table S1) using high mass accuracy, isotopic distribution, and tandem mass spectrometry. The detected species were mostly deprotonated small metabolites and lipids including free fatty acids, cholesterol sulfates, cyclic phosphatidic acids, and glycerophospholipids. Some typical molecular characterization data using collision-induced dissociation (CID) are presented in Fig. S1. The metabolic changes over the time course were more prominent in kidneys subject to UUO than those observed in the sham controls (Fig. 1). Indeed, a remarkable change of ion signals in the *m/z* range 700–900 distinguished UUO from sham control at every time point after obstruction.

### Visualizing the distribution of metabolites and lipids on kidney tissue sections

**Figure 2:**
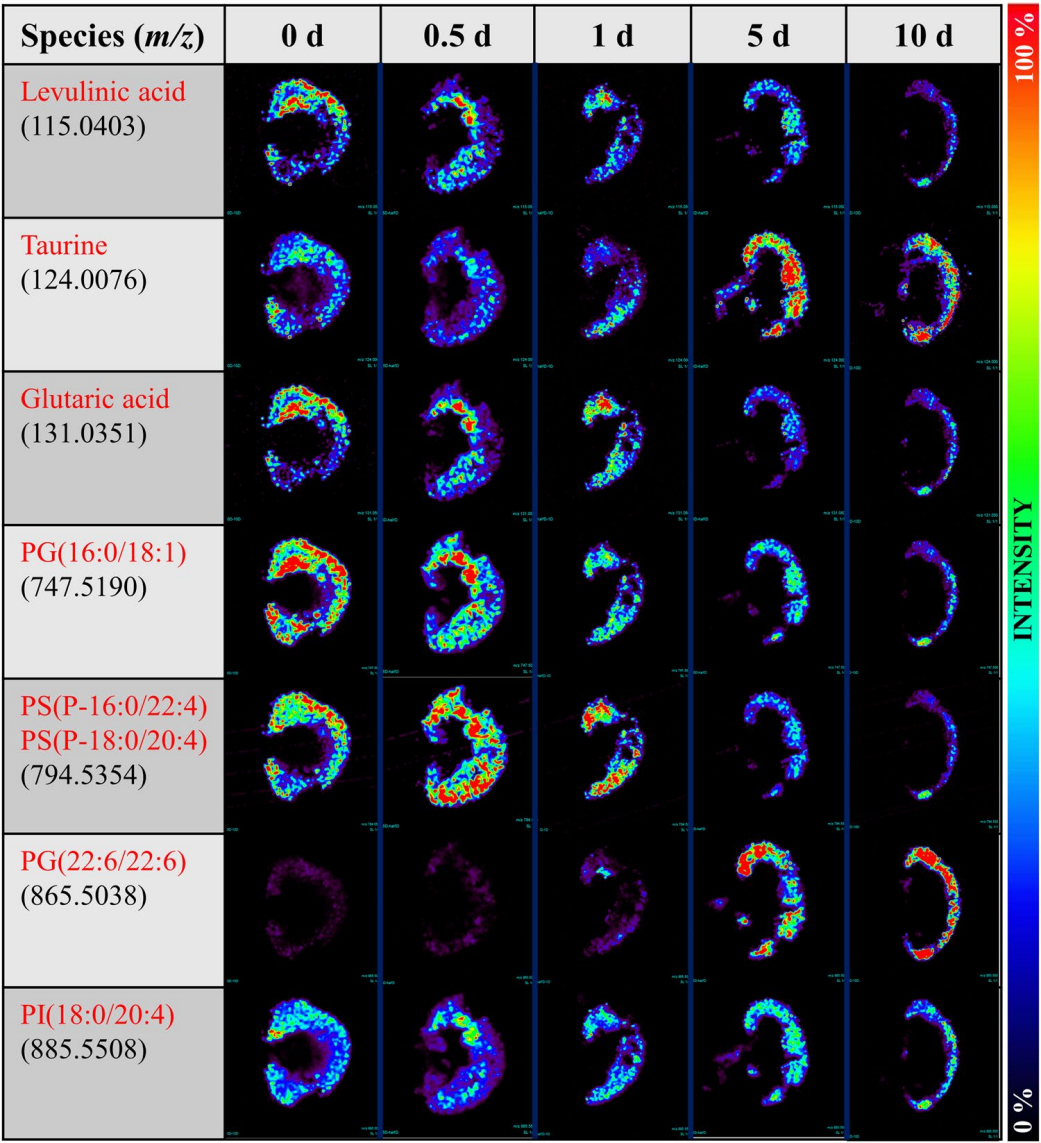
Temporal profiles of ion images in UUO. DESI-MS imaging showing the spatial distribution of 7 different metabolites and lipids in the kidney tissue specimens after UUO of a group of 5 mice sacrificed at five different time points, recorded in days.

We performed negative ion mode DESI-MSI to map the distribution of more than 100 different molecular species throughout the kidney sections to monitor the progress of changes after obstruction at a resolution of ~200 µm. Time-dependent distributions of some typical small metabolites and lipids in the post UUO kidney specimens are displayed in Fig. 2. Although DESI-MSI enables mapping of many small molecules simultaneously, it is limited to those molecular species that are ionized in the gas phase. Because polar protic (acidic) metabolites and lipids can be ionized and imaged generally by negative ion mode DESI-MSI under normal conditions^22^, we were restricted to intercepting those species to correlate with the obstruction over the time course. Despite this restriction, a number of ion images of small metabolites/lipids in Fig. 2 appeared to discriminate renal specimens collected at different time points after UUO. For example, the amount of levulinic acid, glutaric acid, and PG(16:0/18:1) all decreased after UUO and this decrease appeared to be restricted to the renal cortex, whereas taurine and PG(22:6/22:6) both increased over time. Interestingly, the ion image of *m/z* 794.5354, which corresponds to the isomeric mixture of two acylglycerophosphoserines, PS(P-16:0/22:4) and PS(P-18:0/20:4), increased at 0.5 d and then decreased at later time points. Based on their relative abundances, the spatial distribution of many additional intense ion signals (*m/z* 885.5508, 327.2334, 303.2327, 281.2483, 279.2338, 255.2327) did not appear to differ significantly between UUO and sham kidneys over the time course.

### Metabolic markers altered after UUO

**Figure 3:**
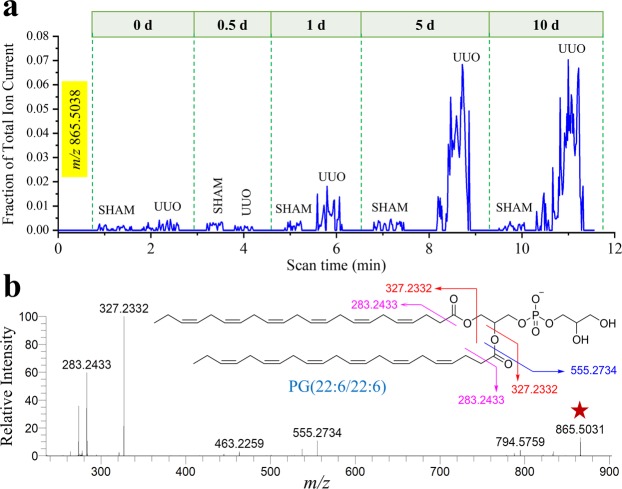
Identifying the potential lipid marker for UUO. (**a**) Extracted ion chronogram of *m/z* 865.5038 (normalized with the total ion abundance) over sequential scribble scanning by DESI-MS of representative five pairs (sham control and UUO) of kidney tissue specimens at five different time points. (**b**) Collision-induced dissociation (CID) of *m/z* 865.5038 (Table S1), marked with a red star, identifies the species as PG(22:6/22:6). Inset of *B* shows the gas-phase fragmentation profile of the species identified.

Given a large number of small molecules (*m/z* 50–1000) interrogated, the pixel-to-pixel mass spectral data obtained from DESI-MSI analysis allowed us to select specific metabolites/lipids (Fig. 2) as potential markers of renal injury from UUO. Because pixel-by-pixel scanning across the tissue is time-consuming (typically 30 min per kidney section), we employed a rapid scribble scanning method to record ion signals from a renal specimen within 1 min (see Experimental section for details). Data obtained from this scribble scanning method reconciled well with that of the whole tissue imaging. For example, based on the ion images, we identified a peak (*m/z* 865.5038) that was sharply upregulated with time. Scribble scanning of sham control and UUO renal specimens over five different time points allowed us to quantify levels of this metabolite (Fig. 3a) and show that the levels reflected those obtained by DESI-MSI (Fig. 2). CID of this species showed that the molecular structure was phosphatidylglycerol PG(22:6/22:6) (Fig. 3b). The rapid rise of the PG(22:6/22:6) ion signal at 1 d after UUO compared to sham controls indicated that it could be a promising early lipid biomarker to detect incipient renal damage since our previous work has shown that there are no histological changes observable by 1 day^7^. Extracted ion chronograms (Fig. S2) from scribble scanning of 8 kidney sections at each time point suggested similar trends of many other ion abundances for both small metabolites (Fig. S3) and glycerophospholipids (Fig. S4) which could be used to distinguish UUO from sham control.

For molecular species with very low abundance in the pixel-to-pixel mass spectral data, it is not possible to construct MS images to visualize candidate markers of renal injury. However, rapid scribble scanning allowed extraction of high-quality signals of those species based on ion chronograms (Fig. S5), allowing us to detect their relative abundance in UUO and sham control groups. Ion chronograms obtained from scribble scanning of replicates for each time point showed increased levels of several important small metabolites including pyruvate, lactate, fumarate, succinate, malate, and glutamate over the time course, particularly after 1 d, in UUO kidneys compared to the corresponding sham controls.

**Figure 4:**
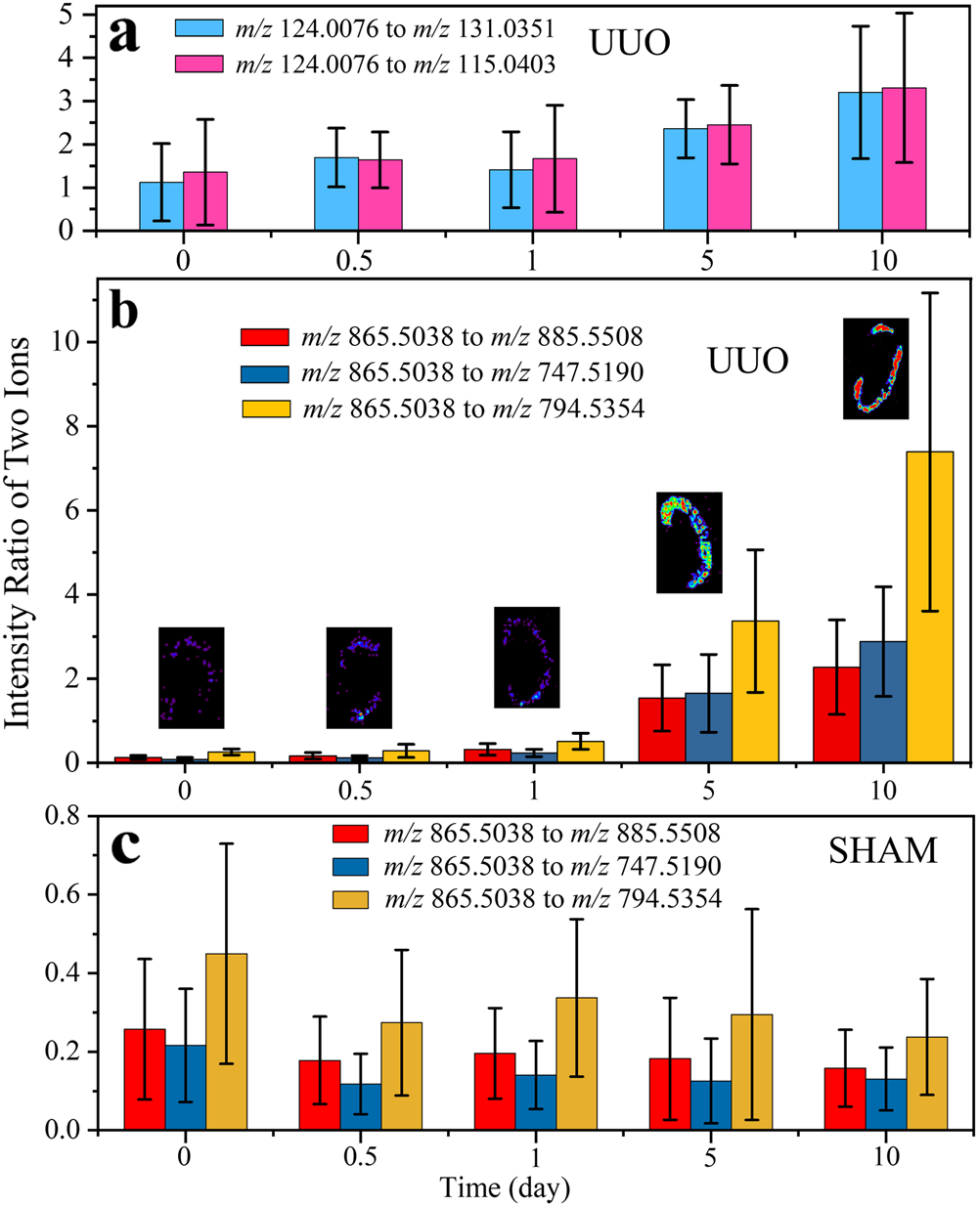
The intensity ratio of two ions revealing the diagnostic features. Negative ion mode DESI-MS ion signal intensity ratios for **(a**) small metabolites, taurine (*m/z* 124.0076) to glutaric acid (*m/z* 131.0351) and levulinic acid (*m/z* 115.0403) at different time points after UUO. Similar signal intensity ratios for glycerophospholipids, PG(22:6/22:6) [*m/z* 865.5038] to PI(18:0/20:4) [*m/z* 885.5508], PG(16:0/18:1) [*m/z* 747.5190], and PS(P-16:0/22:4)/(P-18:0/20:4) [*m/z* 794.5354] at different time points are also presented for (**b**) UUO and (**c**) the corresponding sham control. Inset ion images of *B* show the distribution of *m/z* 865.5038 to *m/z* 794.5354 intensity ratio for the representative kidney tissue at each time post UUO. The data at each time point of each panel represent the mean ± SD calculated from eight mice.

Previously, we found that the ratio of two ion signals could better distinguish between tissue states (normal vs. malignant), and therefore explored whether the intensity ratios of two metabolites could be used to monitor the progression of renal injury caused by UUO. We observed a continuous increase of taurine/glutaric acid and taurine/levulinic acid ion signal ratios in UUO over the time course (Fig. 4a), whereas those ratios remained almost unaltered with time in the corresponding sham controls (data not shown). Interestingly, the ion signal intensity ratios for PG(22:6/22:6) to PI(18:0/20:4), PG(16:0/18:1) and PS(P-16:0/22:4)/(P-18:0/20:4) over the time course for the UUO and sham control kidneys, showed continuous increases in these ratios in UUO (Fig. 4b), while these ratios decreased slightly in the sham controls (Fig. 4c). The 2D distribution of the intensity ratio of *m/z* 865.5038 to *m/z* 794.5354 can be used to generate an image that visually displays the relative changes in the intensity of PG(22:6/22:6) to PS(P-16:0/22:4)/(P-18:0/20:4) at each time point (inset, Fig. 4b).

### Identification of metabolites for early detection of UUO

Although visual analysis of 2D ion images and extracted ion chronograms can be used to identify some important metabolite and lipid markers of renal injury after UUO, the previous work^11,13,14,23^ suggested that simultaneous examination of metabolites and lipids, captured by DESI-MS from the tissue surface, could provide better predictive models. Since we were interested in identifying metabolite changes early in the time course, before histological changes such as renal scarring were apparent, we compared UUO to sham control samples at the 12 hours (0.5 day) time point. Using a set of 48 abundant metabolites/lipids measured well by rapid scribble scanning of UUO and sham control kidneys (Table S1), we applied SAM and the LASSO/Logistic methods at time point 0.5 days, to discriminate UUO from sham controls. We identified 20 peaks that could be used to accurately distinguish UUO from controls with a high degree of accuracy (94%) (Table S2). Similarly, we applied LASSO to the peaks for each of the remaining time points (1, 5, and 10 days) to distinguish UUO from control kidneys. Table 1 presents the results showing the accuracy of the statistical prediction for each of the time points. We achieved 69%, 100% and 94% prediction accuracy at 1 d, 5 d, and 10 d, respectively.

**Table 1:** Statistical prediction results for sham-operated and UUO kidneys at different time points^#^.

Actual	Statistical Prediction							
	0.5 d		1 d		5 d		10 d	
	SHAM	UUO	SHAM	UUO	SHAM	UUO	SHAM	UUO
SHAM	7	1	5	3	8	0	8	0
UUO	0	8	2	6	0	8	1	7
Overall agreement	93.75%		68.75%		100%		93.75%	

^#^DESI-MS data from eight pairs (UUO and corresponding sham control) of kidney tissues at each time point were classified using metabolites identified by comparing UUO to control at 0.5 days using SAM (significance analysis of microarrays) and the Lasso/logistic methods.

### Lipid metabolic pathways are significantly altered after UUO

**Figure 5:**
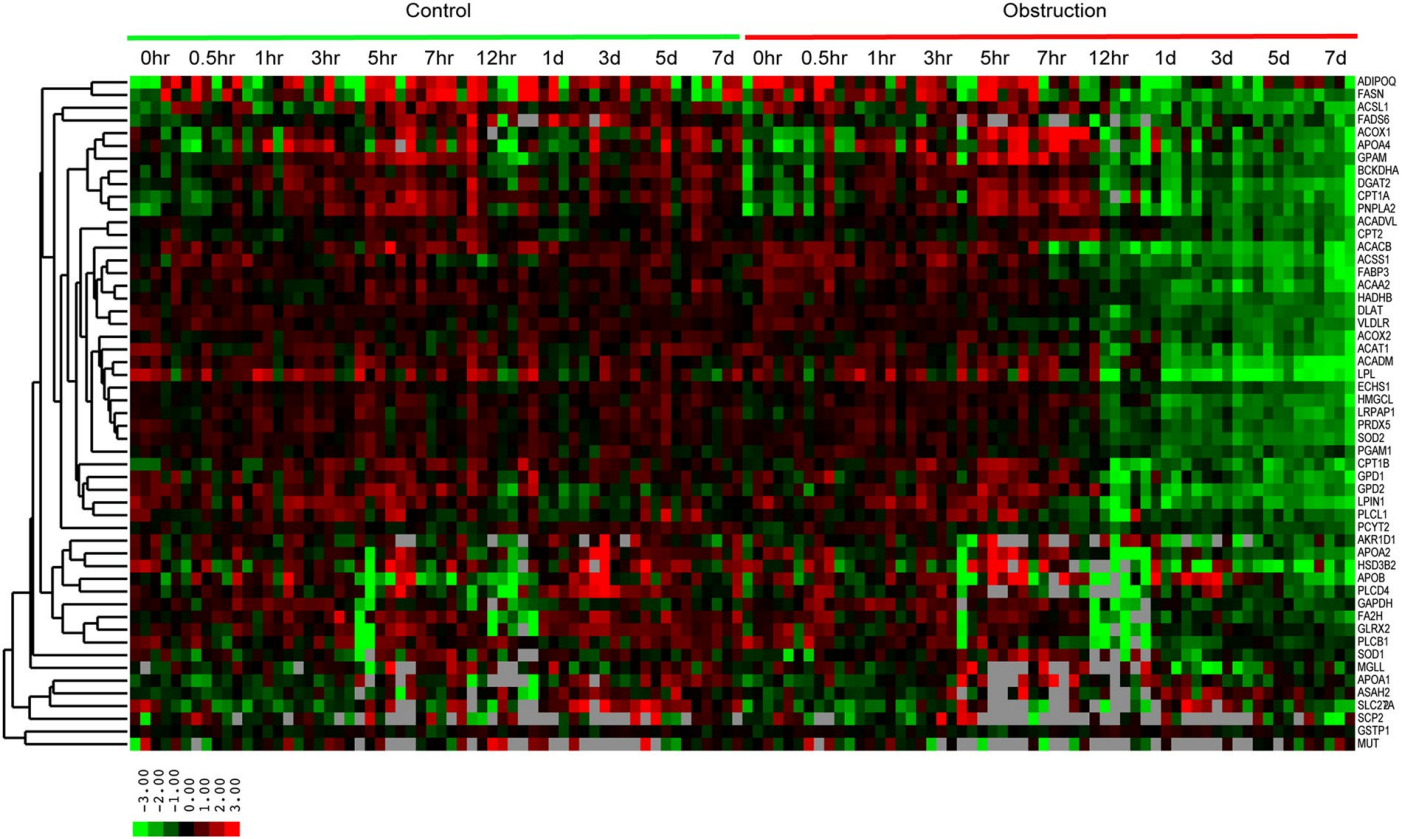
Transcript profiles of genes involved in lipid metabolism in the kidneys of sham controls and mice undergoing UUO over a time course. Transcripts were selected from the literature from IPA as involved in lipid metabolic pathways. Expression levels are median centered, and the degree of color saturation correlates with expression levels. Red indicates higher level expression and green lower. Note decreased expression of nearly all lipid metabolic genes induced by 12 hours post UUO and persisting to 7 days.

A major portion of the metabolites that changed after UUO were lipids and glycerophospholipids (Table S1). Previously, we analyzed gene expression profiles after UUO from a time course experiment that had been performed in the same mouse strain^8^. Using a set of 1084 transcripts modulated significantly over a time course in UUO compared to sham-operated kidneys, we performed Ingenuity Pathways Analysis (IPA). When all genes were considered, IPA gene expression networks can be associated with cell-to-cell signaling, molecular transport, cellular transport, injury, inflammatory response and lipid metabolism (Table S3). Virtually all of the genes identified as significantly associated with lipid metabolism were down-regulated over the time-course after UUO. When up-regulated genes were analyzed separately, only 2 lipid metabolic genes, SGPL1 and SPNS2, were identified. Analysis of down-regulated genes alone showed that lipid metabolism was the top network modulated after obstruction (Table S4). Altered gene expression could be seen by 12 hours after obstruction for several genes, paralleling the time course of lipid and glycerophospholipid changes observed (Fig. 5). Down-regulated genes that we identified included those involved in glycerophospholipid synthesis, including GPAM and GPD1.

## Discussion

DESI-MS provides rapid, an in-depth assessment of metabolic changes after injury, revealing metabolic shifts that occur at time points well before morphological changes and inflammatory infiltrates are visible histologically in this model system. These metabolic changes parallel early and broad-based changes that occur in transcript levels of multiple genes involved in lipid metabolism. Using well-measured metabolites that could be obtained rapidly (<1 min), we were able to construct a model that predicted kidney injury early in a time course, even when using a modest number of samples. Therefore, DESI-MS could have applications in the management of benign renal conditions and should be explored, much as it is being explored in oncology, as a possible adjunct to histology in analyzing renal tissue biopsies from patients.

Broadly speaking, MS approaches have been explored as a source of metabolism-based biomarkers of renal disease in several contexts^5,24,25^. Urine has been the most common biospecimen type analyzed, and a number of metabolites and panels of metabolites that correlate with specific diseases have been identified^5,24–29^. Although some metabolites have been observed across studies, most metabolic changes appear to be poorly reproducible between independent patient sets, different diseases, and different animal model systems. Analysis of blood has shown prediction with several metabolites, particularly some lipids and small metabolites^27,28,30^. Many studies have identified small metabolites, particularly increased levels of products from oxidative phosphorylation in models of, and patients with renal damage. With DESI-MS, we observed a number of these metabolites in the renal tissues of our UUO model including fumarate, succinate, and malate, as well as increases in glutamate, pyruvate, and lactate, which suggested upregulation of the glutaminolysis pathway. Not only did these observations reproduce metabolite findings observed in previous studies of blood urine and tissue, but we were also able to construct a model that achieved nearly 94% accuracy in tissue discrimination (UUO vs sham) as early as 0.5 d after the operation. This remarkably high accuracy in predicting renal damage at an early stage prior to histological evidence of obstruction suggests that DESI-MS could serve as a useful adjunct to histology in assessing renal biopsies.

We found that lipids, particularly glycerophospholipids and lipid metabolic transcripts, exhibited a strong signal in the analysis of renal tissues in our UUO model of renal damage. DESI-MS in negative ion mode using a solvent that preserves histopathology (DMF/ACN) is particularly effective at assessing lipids. Moreover, we found broad-ranging changes in a number of lipids shortly after UUO. Lipids commonly associated with cell membranes, glycerophospholipids, show the most striking changes, and many of these show decreased levels, with PG(22:6/22:6) being a notable exception. These changes in lipid levels are accompanied by early and significant decreases in the transcript levels of many genes associated with lipid metabolism. These include transcripts encoding lipid transporters, metabolic genes, and genes associated with apoptosis. Whether these alterations in lipid levels have functional consequences or are merely reflective of broad metabolic derangements associated with kidney assault is unclear. Lipids do have known functional roles as signaling molecules for inflammation and regulation of perfusion^31^. Forced overexpression of liver-type fatty-acid-binding protein (L-FABP) in the kidney of mice protects against renal inflammation and damage due to UUO, suggesting that lipids and lipid peroxides have a direct role in renal damage^32^.

Given the strong signal observed in the levels and types of lipids altered in response to UUO, it is possible these could be used as biomarkers for renal damage^5,25,33,34^. Previous studies using pulverized kidneys and LC-MS or MALDI-TOF approaches have identified lipid changes in the kidney in several model systems, including UUO. However, these studies show differences in specific lipids altered in response to the type of injury model used. For example, a model of IgA nephropathy showed alterations in triacylglycerols and o-phosphocholines containing 22:6 fatty acids, but with other fatty acid chains that differed from those we observed^35^. While lipids are not typically found in urine, alterations in plasma lipid levels associated with renal failure have long been described^36^. Whether specific lipids, such as those identified in the kidney tissues, are altered in the plasma lipid components needs to be explored in greater detail. Regardless, the specific lipid profiles associated with different models of renal injury should be explored using DESI-MS in renal tissues.

UUO resulted in a remarkable increase in the level of a phosphatidylglycerol, PG(22:6/22:6) beginning 1 d after obstruction. The significance of this increase is unknown. We have previously observed significant elevations of PG(22:6/22:6) in an MYC-driven mouse model of renal cell carcinoma that relies on a kidney-specific promoter with a tetracycline responsive element^19^ and a mouse lung cancer model where RAS is conditionally expressed in the lung epithelium. It is possible that PG(22:6/22:6) elevations are induced by proliferation in both model systems. UUO is known to promote the proliferation of renal tubular cells^37^, likely from activation of renal tubular cell repair pathways^38^, as well as inducing MYC and RAS expression^39^. However, increases in PG(22:6/22:6) were not observed in a conditionally driven MYC model of lymphoma implying that there might tissue-specific factors that influence lipid responses^13^. Furthermore, the increases in PG(22:6/22:6) in the RAS lung cancer model were accompanied by broad increases in expression in lipid synthetic genes such as FASN and an increase in docosahexaenoic acid FA(22:6)^17^. However, in our UUO data, docosahexaenoic acid levels decrease, as do nearly all lipid synthetic and metabolic genes. Therefore, the increase in PG(22:6/22:6) might simply reflect decreased liberation of the free FA(22:6) from the glycerophospholipid. Regardless, the causes of the increased level of PG(22:6/22:6) and its functional roles in UUO remain unclear and represent an interesting area for future investigation.

Metabolomic and metabonomic studies of urine in patients with chronic renal failure have demonstrated that decreased urine levels of taurine can serve as a biomarker of renal damage^26^. Urine levels of taurine actually increase significantly after relief of urinary obstruction in patients with acute forms of this condition^40^. Our data demonstrate that tissue levels of taurine increase over time following UUO, and this suggests that taurine becomes sequestered in tissues. Taurine can serve as an anti-oxidant^41^ and treatment of rats with oral taurine after inducing UUO protects against renal damage^42^. Therefore, it is possible that taurine retention has a functional role in protecting against renal damage in acute kidney injury.

In summary, DESI-MS allows for rapid and comprehensive characterization of metabolites in renal tissues in a UUO model of renal obstruction. Changes in the levels of lipids, particularly glycerophospholipids that are often associated with cell membranes, provide strong signals of renal damage that are detectable before histological changes can be observed. These changes parallel changes in gene expression, where a large number of genes associated with lipid transport and metabolism show decreases in expression within hours of UUO. Given these findings, DESI-MS should be explored as a possible adjunct to histology in diagnosing renal damage and etiology. Future DESI-MS studies will focus on whether there are distinct profiles of metabolic changes or ratios of metabolite levels associated with other specific renal diseases in model systems and patient samples.

## Methods

### Chemicals and reagents

All reagents, chemicals, and solvents were obtained from commercial sources unless otherwise stated.

### Animals

Total 80 postnatal day 42 C57BL/6 mice were used in this study. Animal experiments were approved by the Animal Care and Use Committee at Stanford University. Animals were maintained and used according to the *Guide for the Care and Use of Laboratory Animals* (National Research Council 2003). Mice were housed in cages under a controlled temperature 20–40 °C and relative humidity 40–70%, with a 12 h light/dark cycle and fed standard diet and water.

### Induction of unilateral ureteral obstruction in mice

Mice were randomly divided into two sets (UUO set and sham control set), each comprised of 40 mice. The mice kept were anesthetized with ketamine and subjected to UUO for 0 d, 0.5 d, 1 d, 5 d, and 10 d (n = 8 per time point). The left ureter was obstructed with a nontraumatic microvascular clip (5–15 g/mm^2^, 7-mm S&T Vascular Clamp; Fine Science Tools, Foster City, CA) as we have described previously^7,8^. For each time point, eight additional mice underwent laparotomy and dissection of the left ureter without clipping to serve as sham controls.

### Preparation of kidney tissue specimens

Kidney specimens were collected at 0 d, 0.5 d, 1 d, 5 d, and 10 d after the operation for both UUO and sham control. The kidneys were excised and blood was washed off using saline perfusion rapidly followed by snap freezing and storing at −80 °C until sectioning and analysis. The frozen kidneys then were embedded in a minimal amount of OCT compound and sectioned at 15-µm thickness using a Leica CM1950 cryostat (Leica Biosystems). These tissue sections were thaw mounted on glass microscope slides and stored at −80 °C before DESI-MS analysis.

### DESI-MSI study

Detailed methods of tissue imaging by DESI can be found elsewhere^9,22,43,44^. Briefly, a laboratory-built DESI source coupled to an LTQ-Orbitrap XL mass spectrometer (Thermo Scientific) was used for the kidney tissue imaging. Negative ion mode DESI-MSI was performed using −5 kV spray voltage in the *m/z* range 50–1000 with a spatial resolution of 200 μm (spray spot diameter). We selected negative ion mode over positive ion mode because a large number of lipids and small metabolites could be ionized and detected under negative ion mode, as reported previously^22^. Histologically compatible solvent system 1:1 (vol/vol) dimethylformamide/acetonitrile (DMF/ACN) at a flow rate of 0.5 μL/min with a coaxial sheath gas flow (nitrogen at a pressure 170 psi) was used for generating the stream of charged microdroplets. The kidney tissues were scanned under impinging charged droplets using a 2D moving stage in horizontal rows separated by a 200-μm (spatial resolution) vertical step. All imaging experiments were carried out under identical experimental conditions including geometrical parameters, *e.g*., spray incident angle of 55°, spray tip-to-surface distance ∼2 mm, and spray-to-inlet distance ∼5 mm. Data acquisition was performed using XCalibur 2.2 software (Thermo Fisher Scientific Inc.). An in-house program allowed the conversion of the XCalibur 2.2 mass spectral files (.raw) into the image file, which could be read by a biomedical image analysis software called Biomap (freeware, https://ms-imaging.org/wp/). The intensity distributions of different metabolites, lipids, and the ratio of two species were plotted using the Biomap software. We have used rainbow color order in the ion images to represent the highest concentration by red and the lowest concentration by violet.

### DESI scribble scanning

Previously, we have used a rapid scribble scanning method for DESI-MS analysis of prostate tissue specimens, which had the advantage of dramatically shortened scan times and high fidelity for identifying metabolic profiles compared with full image scans^11^. Rapid scribble scanning was carried using similar DESI experimental conditions as described above but instead of scanning in horizontal rows, we scanned the sample by focusing the spray spot randomly onto the tissue specimen intercepting ions across the tissue in less than a minute for each sample. The average mass spectra acquired by this means for each specimen were used wherever applicable.

### Metabolite/lipid identification

The ions in the MS data were identified by searching the MassBank (www.massbank.jp) and the LIPID Metabolites and Pathways Strategy (www.lipidmaps.org/) databases based on high mass accuracy and their isotopic distribution. When the database listed multiple isobaric/isomeric species, we performed collision-induced dissociation (CID) and compared the CID data with that of the standard from the above database to characterize the species wherever applicable. The CID data of the mass selected ions from the kidney tissue specimen are sometimes complex, although the majority of fragment ions matches with those of standards. This complexity could be caused by the interference of isomeric/isobaric ions derived from the biological matrix (tissue). As the position and stereochemistry of the double bond in the fatty acid chain complicate the structural elucidation, they are often tentatively assigned based on the highest probability seen in the database.

### Statistical analysis

XCalibur raw data files (averaged across the scribble scanning) were converted to CSV files for statistical analysis. The raw data in CSV file format were imported to the R language. Although hundreds of metabolites and lipids were detected by DESI-MS, we selected the top 48 peaks (Table S1), whose abundances were significant, and some of which were characterized by tandem mass spectrometry. All average mass spectra acquired by DESI scribble scanning for each time point (8 animals/group and 5 time points) in both UUO and sham control were normalized by the base peak (most intense peak). To determine which metabolic peaks could discriminate UUO from sham control kidneys, we applied the SAM (Significance Analysis of Microarrays) procedure and the LASSO/Logistic methods at time point 0.5 days, using cross validation^45,46^. To evaluation prediction at all time points, we applied the LASSO/Logistic method, using the glmnet R language package.

### Analysis of gene expression data

We analyzed existing gene expression data obtained using Agilent 44 K Mouse Whole Genome Oligonucleotide Microarrays^8^. Specifically, we used a set of 1084 transcripts modulated over a time course of 0 h, 0.5 h, 1 h, 3 h, 5 h, 12 h, 1 d, 3 d, 5 d, and 7 d which were identified using SAM comparing UUO to sham controls. Genes associated with biochemical pathways and function were identified by Ingenuity Pathways Analysis (IPA) and significantly enriched networks identified by comparing UUO to sham controls for all genes, upregulated genes alone and downregulated genes alone (https://www.qiagenbioinformatics.com).

## Supplementary information

Supplementary Information
