# Supplementary material for: Early detection of unilateral ureteral obstruction by desorption electrospray ionization mass spectrometry
Source: Sci Rep. 2019 Jul 29;9:11007. doi: 10.1038/s41598-019-47396-x (PMC6662848; doi:10.1038/s41598-019-47396-x)
Supplement: Supplementary file 1 — Supplementary Information [file 41598_2019_47396_MOESM1_ESM.docx]

***Supporting Information***

**Early detection of unilateral ureteral obstruction by desorption electrospray ionization mass spectrometry**

Shibdas Banerjee^1,2^, Anny Chuu-Yun Wong^3^, Xin Yan^1^, Bo Wu^3^, Hongjuan Zhao^3^, Robert J. Tibshirani^4^, Richard N. Zare^1,^*, James D. Brooks^c,^*

^1^Department of Chemistry, Stanford University, Stanford, CA 94305, USA

^2^Department of Chemistry, Indian Institute of Science Education and Research Tirupati, Tirupati 517507, INDIA

^3^Department of Urology, Stanford University School of Medicine, Stanford, CA 94305, USA

^4^Departments of Biomedical Data Sciences, and of Statistics, Stanford University, CA 94305, USA

^*^Correspondence to James D. Brooks and Richard N. Zare; Emails: [zare@stanford.edu](mailto:zare@stanford.edu) and [jdbrooks@stanford.edu](mailto:jdbrooks@stanford.edu)

**Table S1.** List of abundant ions in the mass spectra data included in the statistical analysis.

| *Sl. No.* | *Measured*  *m/z** | *Attribution^#^* | *Theoretical m/z^†^* | *∆m/z* | *Error*  *(ppm)* |
| --- | --- | --- | --- | --- | --- |
| 1 | 66.1552 | n.d. |  |  |  |
| 2 | 89.0247 | Lactic acid | 89.0244 | 0.0003 | 3.36986 |
| 3 | 101.0247 | 2-Ketobutyric acid | 101.0244 | 0.0003 | 2.96958 |
| 4 | 115.0403 | Levulinic acid | 115.0401 | 0.0002 | 1.73852 |
| 5 | 124.0076 | Taurine | 124.0074 | 0.0002 | 1.61281 |
| 6 | 131.0351 | Glutaric acid | 131.0350 | 0.0001 | 0.76315 |
| 7 | 152.9960 | n.d. |  |  |  |
| 8 | 155.0714 | 5-oxo-7-octenoic acid | 155.0714 | 0 | 0 |
| 9 | 168.0076 | n.d. |  |  |  |
| 10 | 183.0129 | n.d. |  |  |  |
| 11 | 224.0337 | n.d. |  |  |  |
| 12 | 242.0443 | 1-alkyl-2-acylglycerophosphoethanolamine. | 242.0435 | -0.0008 | -3.30519 |
| 13 | 254.9792 | n.d. |  |  |  |
| 14 | 255.2327 | FA(16:0)/Palmitic acid | 255.2329 | -0.0002 | -0.7836 |
| 15 | 273.8128 | n.d. |  |  |  |
| 16 | 279.2338 | FA(18:2) | 279.2330 | 0.0008 | 2.86499 |
| 17 | 281.2483 | FA(18:1)/Oleic acid | 281.2486 | -0.0003 | -1.06667 |
| 18 | 283.2640 | FA(18:0) | 283.2643 | -0.0003 | -1.05908 |
| 19 | 303.2327 | FA(20:4)/arachidonic acid | 303.2329 | -0.0002 | -0.65956 |
| 20 | 311.1696 | *N*-Undecylbenzenesulfonic acid | 311.1686 | -0.001 | -3.21369 |
| 21 | 325.1852 | 4-Dodecylbenzenesulfonic Acid. | 325.1843 | -0.0009 | -2.76766 |
| 22 | 327.2334 | FA(22:6) | 327.2330 | 0.0004 | 1.22237 |
| 23 | 419.2579 | CPA(18:0) | 419.2568 | 0.0011 | 2.62369 |
| 24 | 465.3054 | Cholesterol sulfate | 465.3044 | 0.0010 | 2.14913 |
| 25 | 480.2742 | n.d. |  |  |  |
| 26 | 508.3057 | n.d. |  |  |  |
| 27 | 528.2742 | n.d. |  |  |  |
| 28 | 552.2743 | n.d. |  |  |  |
| 29 | 556.2693 | n.d. |  |  |  |
| 30 | 580.2692 | n.d. |  |  |  |
| 31 | 594.3061 | n.d. |  |  |  |
| 32 | 606.3425 | n.d. |  |  |  |
| 33 | 622.3374 | n.d. |  |  |  |
| 34 | 646.6150 | Cer(d18:1/24:1(15Z)) | 646.6144 | -0.0006 | -0.92791 |
| 35 | 665.4049 | n.d. |  |  |  |
| 36 | 681.3998 | n.d. |  |  |  |
| 37 | 722.5149 | PE(P-16:0/20:4) | 722.5130 | 0.0019 | 2.62971 |
| 38 | 747.5190 | PG(16:0/18:1) | 747.5182 | 0.0008 | 1.07021 |
| 39 | 766.5407 | PE(20:4/18:0) | 766.5392 | 0.0015 | 1.95685 |
| 40 | 773.5356 | PG(20:2/16:0) | 773.5338 | 0.0018 | 2.32698 |
| 41 | 794.5354 | PS(P-16:0/22:4),  PS(P-18:0/20:4) | 794.5341 | 0.0013 | 1.63618 |
| 42 | 810.5294 | PS(18:0/20:4) | 810.5291 | 0.0003 | 0.37013 |
| 43 | 817.5042 | PG(18:2/22:6) | 817.5025 | 0.0017 | 2.0795 |
| 44 | 841.5042 | PG(20:4/22:6) | 841.5025 | 0.0017 | 2.0202 |
| 45 | 857.5192 | PI(16:0/20:4) | 857.5186 | 0.0006 | 0.69969 |
| 46 | 865.5038 | PG(22:6/22:6) | 865.5025 | 0.0013 | 1.50202 |
| 47 | 885.5508 | PI(18:0/20:4) | 885.5499 | 0.0009 | 1.01632 |
| 48 | 909.5516 | PI(18:0/22:6) | 909.5499 | 0.0017 | 1.86906 |

*****High mass accuracy/mass resolution measurements were obtained using an Orbitrap mass spectrometer. Experiments conducted in different days provided *m/z* value of the same ion signal with slight variation in its third or fourth decimal places because of the variation of calibration. This table lists the *m/z* values after the best possible calibration of the instrument during this study.

^#^Tentative assignments, wherever possible, are based on high mass accuracy, isotopic distribution, and tandem mass spectrometry (using the collision-induced dissociation technique). FA, fatty acid; CPA, cyclic phosphatidic acid; Cer, Ceramides; PA, phosphatidic acid; PG, glycerophosphoglycerols; PE, glycerophosphoethanolamine; PS, glycerophosphoserines; PI, glycerophosphoinositols. (M:N/X:Y) denotes the number of carbons (M and X) and the number of double bonds (N and Y) in each of the two fatty-acid chains. As the position and stereochemistry of the double bond in FA complicate the structural elucidation, they are often tentatively assigned in FA and glycerophospholipids (GP).

^†^Theoretical *m/z* was based on the monoisotopic mass of the respective deprotonated species.

n.d., not determined (unassigned)

**Table S2.** List of top 20 features (*m/z* values) along with their t-statistic for UUO *vs* sham control^ǂ^, from a SAM analysis

| *Sl. No.* | *m/z* | *t-statistic* |
| --- | --- | --- |
| 1 | 766.5407 | -6.331862 |
| 2 | 885.5508 | -6.135255 |
| 3 | 283.2640 | -5.073995 |
| 4 | 747.5190 | -4.969935 |
| 5 | 773.5356 | -4.956035 |
| 6 | 794.5354 | -4.90668 |
| 7 | 622.3374 | -4.312275 |
| 8 | 665.4049 | -3.823668 |
| 9 | 419.2579 | -3.676367 |
| 10 | 681.3998 | -3.552273 |
| 11 | 327.2334 | -3.543751 |
| 12 | 131.0351 | -3.273319 |
| 13 | 810.5294 | -3.241524 |
| 14 | 480.2742 | -3.203015 |
| 15 | 168.0076 | -3.061601 |
| 16 | 857.5192 | -2.930769 |
| 17 | 817.5042 | -2.899778 |
| 18 | 303.2327 | -2.892302 |
| 19 | 255.2327 | -2.864096 |
| 20 | 419.2579 | -3.676367 |

**^ǂ^**The higher the negative value of t-stat, the lower the probability of UUO.

**Table S3.** Ingenuity Pathway Analysis (IPA) gene expression networks and functions of 1084 transcript significantly modulated after UUO compared to sham controls

| Rank | Molecules in Network | Score | Focus  Molecules | Top Diseases and Functions |
| --- | --- | --- | --- | --- |
| 1 | ARG1, BET, C1QTNF3, CORO1A, CPEB1, ELF3, F13A1, FGA, FGG, FMO3, FUT9, G6PC, Havcr1, HSD11B1, HSPB1, HSPB8, IL6, NCF4, NEK6, NFKBID, OSMR, P2RY6, PDK4, PEPCK, PGLYRP2, PILRA, PILRB, PRKAA, PTPN22, SERPIND1, ST3GAL1,  STX11, TNFR, TNFAIP8, WFDC17 | 34 | 31 | Cell-To-Cell Signaling and Interaction, Hematological System Development and Function, Immune Cell Trafficking |
| 2 | AQP2, AQP3, AQP4, AQP5, ASNS, AVPR2, Cdc2, CEBPB, CKAP2, FOS, GLIPR2, HIST1H3A, HIST1H4, HIVEP3, HLF, INMT, KRT14, LCN2, LHX5, MMP14, NDRG4, NFKBIZ,  PCTP, RAP1GAP, RNA POL2, S100A8, S100A9, SAA3, SLC16A6,  SLC20A1, SLC7A1, TEF, TMSB4X, TRPV6, UHRF1 | 34 | 31 | Molecular Transport, Cell Morphology, Developmental Disorder |
| 3 | AACS, ADRB, AKR1C14, ANKRD1, ASS1, CD59, CD200R1, CSF3R, CXCL16, CYFIP2, CYP51A1, FGL2, GDF15, GSTM1, HGB, ICAM1, IFNGR2, IL10RA, INHBB, INF alpha, IRF1, ITGB7, KDR, KLK3, LHX1, LRG1, MLKL, PDGFB, RGS18, RRAS, RSAD2, Spink12, SRXN1, SYCN, VEGF | 34 | 31 | Cell-To-Cell Signaling and Interaction, Hematological System Development and Function, Inflammatory Response |
| 4 | ADAMTS1, ADORA2B, BDKRB1, CASP4, CCL17, CD300LF, CSTB, CXCR4, EDN1, FGF18, GAL3ST1, GPR34, HAS1, HDAC, IER2, IL1B, ITPR, MEFV, MEK, MEOX1, MEOX2, MYO5A, PDE4, PDE4B, PLAC8, PLSCR1, PVR, SEMA3C, SERPINA3, SLC12A1, SLCO1A1, SORL1, TLR, UGCG, WNT9B | 32 | 30 | Cellular Movement, Metabolic Disease, Organismal Injury and Abnormalities |
| 5 | ADAM8, CTNNA1, Ccl2, CCL4, CCL5, Ccl7, Ccl8, Ccl9, CCR2, CCR5, CD300A, chemokine(s), CIAP, COCH, CXCL2, CXCL6, CXCL12, EHF, F3, Fcer1, FGR, FZD9, IFI44, IFNGR1, IL36A, LITAF, MYBPC2, PLAUR, PTX3, SRC (family), SRPX2, TNFAIP3, TNFRSF12A, TNNI3, TREM2 | 32 | 30 | Cellular Movement, Hematological System Development and Function, Immune Cell Trafficking |
| 6 | ANGPT2, B2M, BCL3, CD8, CD44, CD8B, CEBPD, CLU, CNKSR3, CTSC, CXCL3, CCNA, E2F, GADD45A, IFI16, IL1R2, ITGA6, ITGAX, ITM2A, JUN, LCK, LY6D, MAP2K1/2, MMP3, MMP7, PLK3, PROX1, RB1, SDC1, SLC5A8, SPRR1A, TEK, TG, TNFRSF1B, TTR | 32 | 30 | Tissue Development, Cancer, Organismal Injury and Abnormalities |
| 7 | AMPK, ATG7, ATP8A2, BDH1, CD244, CDCA7, CDKN1A, CLCA1, CLDN1, CLEC7A, COX6A2, CYCS, EGR1, EGR3, EMP1, ERG, IKZF1, IL2RB, INSC, IVL, LH, MUC(s), MYC, NOTCH1, NR2E3, PODXL, RND3, RUNX1, SLC5A9, SOX4, SOX9, STAG3, TES, TNFRSF19, Ubiquitin | 32 | 30 | Cellular Development, Cellular Growth and Proliferation, Hematological System Development and Function |
| 8 | Ank2, ANXA2, APOC2, ARG2, BBC3, BMP6, C5, CCL2, Ccl6, FCGR1A, FCGR2A, GPR65, HP, IFN alpha/beta, IL1, IL15, Integrin, Klra7, KLRB1, LTB, NCKAP1L, Nr1h, P38 MAPK, PTPRE, RAS homolog, RGS1, RHOB, RTN4, S100A10, SBNO2, SLC4A1, SLC8A1, THEMIS2, TREML4, TSC22D1 | 30 | 29 | Organismal Injury and Abnormalities, Respiratory Disease, Infectious Diseases |
| 9 | ALDH1A2, B4GALNT1, C3, C3AR1, C4A/C4B, C4BP, CFP, CLEC4M, COL1A1, COL5A1, COX1/2, CYR61, Fibrin, GZMA, JNK, KLF6, KRT8, KRT19, KRT20, MAP3K1, MRC1, NGFR, NR4A1, PKC(s), PROZ, SERPINE1, SPON1, SPP1, SPTLC2, TGFBI, TIMP1, TNN, USP2 | 30 | 29 | Organismal Survival, Endocrine System Disorders, Gastrointestinal Disease |
| 10 | ACAT2, AKR1B1, CD84, CHRNA4, CLEC4D, COL4A1, CPT1, CPT1A, CTSS, FLOT1, GADD45B, GCGR, GJB3, HDL-cholesterol, IGG, IGM, IRS1, LDL-cholesterol, LEPR,LPL, MOGAT2, Mt1, Mt2, P2RY13, PLVAP, RGS16, RHOU, SFN, SLC2A4, SLC9A3, TNFRSF13B, TRIB1, UBIAD1, VLDL-cholesterol, VPREB1 | 30 | 29 | Protein Synthesis, Humoral Immune Response, **Lipid Metabolism** |
| 11 | ACSL4, AKR1B10, AQP11, ATF3, BHMT, CCK, COX7A1, CX3CL1, CYBB, DUSP10, EGF, EPHA2, ERK, ERK1/2, GCH1, HMOX1, IL12 (family), IL1RN, LAT2, MMP8, NADPH oxidase, NCF2, Nrg1, PI3K (complex), PIK3R1, PPP1R1B, PTGER1, RAS, RSK, S100A6, THRB, TLR2, TLR6, TRIB3, TRPM2 | 28 | 28 | Cell Death and Survival, Organismal Injury and Abnormalities, Cancer |
| 12 | AREG, BIRC3, CCL20, CCL21, CD83, CH25H, CLEC4A, Col17a1, CXCL10, EFHD2, IFNAR, IGA, IGG1, IGG3, IGG2a, IGG2B, IL12 (complex), MHC Class II, MS4A4B, NFKB2, NPY1R, P2RY10, PLCG1, PLK, RASGRP3, REG3G, RRAD, SAMSN1, SCARA3, SERPINB5, SPIB, TBXA2R, TNFAIP6, VCAM1, ZC3H12A | 23 | 25 | Humoral Immune Response, Protein Synthesis, Hematological System Development and Function |
| 13 | AP1, ARID5A, BATF, CD3, COL2A1, COLI, CXCR3, DSG2, DUSP2, EFEMP2, EGR2, EPOR, F10, FGB, GC, Gsk3, JUNB, LGALS3, LRP2, MAPK, MCL1, MSR1, NFAT, OAS2, PTPRC, SAA, SERPINA3G, SOCS2, STAT3, STAT5A/B, TCR, TEC, THY1, TNXB, VAV | 22 | 24 | Inflammatory Response, Organismal Injury and Abnormalities, Cancer |
| 14 | AKAP12, AKT, APBB1IP, BCL10, BCL2A1, BCR (complex), BTG2, BTK, CD2AP, CCNB1, FCGR, FYB1, HBEGF, HCK, HCLS1, IKK (complex), IGG, ITGAM, ITGAV, LCP2, MAP3K14, PDGFR, PIK3R5, PKG, RALA, Rap1, RHBDF2, SERCA, SIRPA, SYK/ZAP, TLR1, TYROBP, VASP, WAS | 20 | 23 | Hematological System Development and Function, Tissue Morphology, Cell Morphology |
| 15 | 26s Proteasome, ALT, BCL11A, Casp12, CD14, CK, FOXJ1, GCNT2, GOT, HAVCR2, HIVEP2, HSD3B1, IFN Beta, IFN type 1, IKB, IL-1R, IL18R1, IL1RL2, KRT18, NFkB (complex), NFKBIE, PKA, PPP1R1A, RAC, RIPK3, Rock, SAA1, SFTPD, SOCS3, SPHK1, STAT4, TAGLN, TRAF1, TRIM63, ZBP1 | 20 | 23 | Hematological System Development and Function, Lymphoid Tissue Structure and Development, Tissue Morphology |
| 16 | ACY1, ADAMTS2, AHR, ALDH9A1, ARG2, ASPN, BCR (complex), C11orf86, CHRNB1, COL11A1, COLQ, CORIN, DOK7, ITK, KLRC1, Klrc2/Klrc3, KLRD1, KMT2D, LTBP3, MPEG1, MS4A6A, MYO18B, MYO1F, PCSK6, PDE4C, PPARD, PRDM1, PRR15, RAPSN, RELL1, SLC16A5, SLC6A12, SORD, ST8SIA6, TRAF3 | 19 | 22 | Developmental Disorder, Hereditary Disorder, Immunological Disease |
| 17 | Acot1, ATF3, BTNL2, CA3, CXCL16, F3, FAM107A, FGR, GBP4, GBP6, GPRC5B, HOXA10, IFNG, IKZF2, IL11, ITIH5, KLF10, PCDH17, PCDHB16, PFKFB3, PIGR, PIK3R2, PSCA, PTEN, SDC1, SFTPD, SHD, SLC15A3, SPRR1A, ST14, STAG1, STEAP1, TLR6, TMEM64, UBD | 17 | 21 | Infectious Diseases, Cellular Function and Maintenance, Cell Death and Survival |
| 18 | AHR, AHRR, AIP, APP, ARG2, BFSP2, CAMK2N2, CD274, CHD7, CTSS, EDN1, FBN2, FOSL1, GALNT11, GCA, GPRC5A, HSP90AA1, HSP90AB1, IER3, IL-1R, ITM2B, LGALS1, NFKBIZ, PCDH8, PDE4B, PDE8A, PMAIP1, S100A8, S100A9, Saa3, SERPINA7, SYT12, TLR3, ZFP36 | 16 | 20 | Cancer, Organismal Injury and Abnormalities, Renal and Urological Disease |
| 19 | ADAM8, ADAM11, ADAM19, ANKRD6, CADM1, CCL24, CD300LF, DGKA, DUSP10, EPHX1, FOXP1, GNA15, HIPK2, HLX, IER2, IL2, IL4, KCNT1, MAP3K2, NUP155, PDLIM7, PIM2, PLSCR1, PSTPIP1, RASD1, RASGRP4, S100A10, SERPINB6, SRF, STAT6, STC2, TLN2, TMEM40, UPK3B, USP2 | 16 | 20 | Cell Morphology, Hematological System Development and Function, Lymphoid Tissue Structure and Development |
| 20 | ABCA13, ACOT2, BCKDHB, CEBPE, CLDN1, CLDN4, CLDN6, CRHR2, DKK3, Ear2, FGF9, GATA2, GYPA, HHEX, KCNK9, KEL, LCN2, LYPD3, Mcpt4, MTHFD1, MTHFD2, PIK3C2G, PSD3, RETNLG, RHAG, RHCE/RHD, SLC34A2, SMOX, SPARC, SPRR1A, SPRR2G, STFA1, TMC5, UCP1, VEGFA | 16 | 20 |  |
| 21 | Acot1, ASNS, ATP7A, BDH1, C11orf54, CBR3, CLDN2, CYP7A1, EPS8L3, FMO2, GADD45B, GALM, GSTA5, HNF1A, HNF4A, HSD3B4, LOC102634389, LRP5, Mt1, NKD2, ONECUT1, POR, RETSAT, S100G, SEMA4A, SERPINA1, SERPINA10, SLC10A1, SLC22A18, SLC2A2, SLCO1A1, SLCO1A4, TIMD2, TMIGD1, TMPRSS4 | 13 | 18 | **Lipid Metabolism**, Molecular Transport, Small Molecule Biochemistry |
| 22 | A2M, ANKRD40, ANXA3, AREG, BDKRB2, BTG1, C16orf89, CA5A, CKB, COL18A1, COL4A1, COL4A2, CTPS1, CYP3A7, DAPK1, EFHD1, FBP1, FIGNL1, FKBP4, FOXO1, HBEGF, HSPA9, LIF, NAMPT, NRAP, PTPRU, SGK1, SLC25A5, SLC5A3, SMARCB1, SPTLC2, SUCLG1, TOP2A, TP53, UCHL3 | 13 | 18 | Embryonic Development, Organismal Development, Reproductive System Development and Function |
| 23 | ALP, AMACR, BPNT1, CBFB, CCL4, CCL20, CLEC1A, CREB3L3, CXCR4, DDIT3, DIO2, GAB1, GPM6A, GPR176, IER5, IFI16, IGF2BP2, IL18, IL23A, IL4I1, IRGM, MAFF, MAP3K8, MMP13, ODF4, PTGER4, RARA, RBM3, RNF24, RXRA, SETDB1, SLC16A2, STXBP4, TICAM1, ZFP36L1 | 13 | 18 | Infectious Diseases, Cellular Development, Cellular Growth and Proliferation |
| 24 | ADCYAP1, AR, AREG, ATP4A, BBC3, CA2, CAMK4, CLDN3, COL8A1, CXCL17, CYP17A1, DCDC2, EHD3, ENPP2, ESR1, FGFR2, GAP43, GREM2, INHBB, KRT14, LHB, MAPK7, MUC4, NDUFB2, REN, RICTOR, SCEL, SGK1, SLC26A7, SLC2A12, SMCPO, TAF7L, TMEM158, TP63, UBE3A | 12 | 17 | Embryonic Development, Organ Development, Organismal Development |
| 25 | ADRA2C, ANXA2, ATP1B1, BCL10, BCL2A1C, CD3D, CMTM3, CP, CPNE4, CYR61, DLG4, DPYSL3, DUSP26, E2F5, GMNN, GNAO1, JAZF1, JINK1/2, MAPK1, MAPK8IP3, MIR8, MKNK1, MTORC1, NSMAF, PELI1, PHYHIP, PLEK, PTBP1, RAB7B, RGS16, TLR4, TOLLIP, TSC22D1, TUBB6, VAMP2 | 12 | 17 | Cardiovascular Disease, Organismal Injury and Abnormalities, Renal and Urological Disease |

**Table S4.** IPA gene expression networks and functions of 447 down-regulated genes following UUO compared to sham controls

| Rank | Molecules in Network | Score | Focus  Molecules | Top Diseases and Functions |
| --- | --- | --- | --- | --- |
| 1 | ACACB, AKR1B1, Akr1c14, Akt, Ank2, AQP11, C1QTNF3, COX7A1, CPT1A, CXCL12, EGF, FMO3, G6PC, HDL-cholesterol, HSD11B1, IL15, IRS1, LEPR, LPL, MYO5A, N-cor, Nr1h, PEPCK, PI3K (complex), PPP1R1B, PTGER1, SLC2A4, SLC2A12, SLC8A1, STXBP4, THRB, TNXB, TRPC3, UBIAD1, VEGF | 41 | 28 | **Lipid Metabolism**, Molecular Transport, Small Molecule Biochemistry |
| 2 | AACS, ADRB, ANGPT2, ANKRD1, BDKRB1, BHMT, CCK, CHRNA4, Collagen(s), CYP51A1, EPOR, ERK, ERK1/2, F3, GADD45A, GNAZ, IGG1, IGM, IGG, JNK, NFkB (complex), NPY, NPY1R, P38 MAPK, Proinsulin, PROX1, PTGER3, RALA, RASGRP3, RSAD2, SLC9B2, STAT5A/B, TEK, VPREB1 | 29 | 22 | Nervous System Development and Function, Cardiovascular Disease, Hematological Disease |
| 3 | ASS1, CTNNB1, CYP27B1, DAB1, FOXA1, FUT9, GAL3ST1, GATA3, GATM, HDAC, HNF4A, HOXC8, IL1B, IRX1, IRX2, ISL1, LEP, LHX1, LRP2, MEOX1, MEOX2, MSI2, NKX3-2, POU4F1, POU4F2, PRDM1, S100G, SFRP1, SLC12A1, SLC22A24, SLC5A8, SOSTDC1, STAT3, WNT4, WNT9B | 18 | 16 | Developmental Disorder, Embryonic Development, Organismal Development |
| 4 | ASPA, CCDC117, CREB3L2, CYFIP2, DUSP15, EDNRB, EFHD1, ERG, FHL1, FMO3, FOXO1, G6PC, GFI1B, GNE, GSN, GYS2, HAO2, HNF4A, IGLL1/IGLL5, IL10RA, INHBA, KCNJ11, LMO2, LOC102634389, NEPN, NUDT4, OCLN, PEPCK, PTPDC1, RASGEF1B, SLC22A18, TCF3, TCF7L2, TET2, VPREB1 | 18 | 16 | Cell Morphology, Hematopoiesis, Humoral Immune Response |
| 5 | ALDH9A1, ANGPTL3, AOX3, APOA4, ARNTL, ATP4A, CD8, CORIN, DUSP9, G6PC, GPAM, GSTA5, HSD3B1, IFITM1, IFNB1, IGTP, IL12B, ISGF3, ITM2A, MAPK12, MARK2, NCOA1, NDUFB2, NODAL, OBP2B, PCSK6, PDE4C, PPARD, PROZ, RHBG, RICTOR, RORC, SDHB, SLC16A7, VLDLR | 18 | 16 | **Lipid Metabolism**, Small Molecule Biochemistry, Connective Tissue Development and Function |
| 6 | ACY1, AKT3, BCL11B, BSND, BTNL2, CD74, DNMT3A, EIF4EBP1, ESRRA, FAM107A, FCER1G, FOXP1, GALNT11, GCA, GPRC5B, IFNG, IFNGR1, IGF2R, IL15, ITIH5, KCNT1, LMO2, PCDH17, PDE1A, PEPCK, PGAM2, PIM1, PLAU, PPP1R1B, SCNN1A, SLC2A1, SLC8A1, TCF7, TMEM64, ZFP36 | 16 | 15 | Cellular Function and Maintenance, Hematological System Development and Function, Lymphoid Tissue Structure and Development |
| 7 | AKT3, BBC3, BTG1, CA8, CA5A, CFTR, CKB, CTPS1, DAPK1, DNAJC3, DPYS, DUSP26, GATA1, GLUD1, GSN, GYPA, HIPK2, IDH1, IGFBP2, KRT8, LPIN1, MIR8, MT2, NRAP, OXCT1, PTPRU, RBBP7, SLC4A1, SLC5A3, SLC9A3, SMARCB1, TNFAIP8, TP53, UPK3B, UPP1 | 15 | 14 | Gastrointestinal Disease, Molecular Transport, Dermatological Diseases and Conditions |
| 8 | ABCA13, ADRA2C, AR, CA3, CACNA1G, CCL21, CHST2, COL4A5, CRABP2, CSN2, CTNNB1, DIXDC1, DNAJC6, EHD3, GALM, GATA2, GSTM1, HDAC5, HESX1, HHEX, LHB, LMX1B, MAPK1, MPL, MSI1, MTA2, NUMB, PDYN, PSCA, PTEN, SLCO1A1, SUCLA2, SUCLG1, SUCLG2, TNFRSF19 | 15 | 14 | **Lipid Metabolism**, Nucleic Acid Metabolism, Small Molecule Biochemistry |
| 9 | ACSL4, ADAM8, ALDOC, ANXA2, ASS1, BCKDHB, CCL19, CCL21, CMKLR1, CSF2RB, EPHX1, FGF9, GADD45G, GREM2, HIGD1A, IGHA, IGHG1, IL4, IL5, ITCH, LTB4R, MGEA5, MTHFD1, NR4A3, PRKG1, RAP1GAP, SAA3, SCEL, SCGB1A1, SOCS2, ST8SIA6, TAL2, TLN2, TRAF3, UCP1 | 15 | 14 | Cellular Movement, Hematological System Development and Function, Immune Cell Trafficking |
| 10 | ABCA8A, AKT2, ATF6, CAB39L, CEBPB, CYP11A1, CYP19A1, EHF, EMX1, FBXO32, FGF10, FSH, FST, GLI3, GSK3, HOXA10, HSD11B1, IGFBP1, INMT, IRAK1, KAT2A, KISS1, KLK3, LEPR, MTOR, NDRG4, NKX2-1, NR5A2, PCTP, PLAUR, SHD, SHH, SNCG, SRPX2, SYCN | 15 | 14 | Embryonic Development, Organismal Development, Organ Morphology |
| 11 | ADIPOQ, ALDOB, APP, ARR3, C11orf54, C8A, C8G, CYP19A1, ECH1, FADS6, GNAT2, GTF2IRD1, HLF, HNF1A, HSD11B2, HSD3B1, ITM2B, LRP8, LRRC66, LSS, MSMO1, MVD, NR2E3, NSDHL, OPN1SW, PAH, POR, PPARA, PPP1R1A, PKLR, RETSAT, SC5D, SERPINA3, SLC27A2, SPON1 | 15 | 14 | **Lipid Metabolism**, Small Molecule Biochemistry, Vitamin and Mineral Metabolism |
| 12 | ACTC1, ADAM10, BVHT, CHRDL1, DIO1, DIO2, DLL4, FOXM1, GSK3A, HAND1, INSC, KDR, MAPT, MESP1, MYH6, NCOA1, NTF3, ODF4, PKD1, PLD1, PLVAP, PSEN1, PVALB, SLC16A2, SLC16A4, SLC26A7, SLC8A1, TG, TGFB2, THRB, THRSP, TNNI1, TTR, UNC45B, YY1 | 15 | 14 | Amino Acid Metabolism, Endocrine System Development and Function, Molecular Transport |
| 13 | 14-3-3, ADAM8, ADIPOR1, CTNNA1, AMPK, AQP5, AXL, BFSP2, CNNM1, COCH, COX6A2, CK, EGFL6, ESR2, FOXC2, ITCH, KCNN2, KRT8, LHB, LIPG, NOX1, NPY, OCLN, PAK1, RETN, RGS9, SAA3, SORD, SUSD4, TIMP1, TLR3, TNF, TNFAIP8L2, TNN, USP2 | 13 | 13 | Cellular Movement, Tissue Morphology, Immune Cell Trafficking |
| 14 | ALP, AMACR, ASCL1, BMPR2, BPNT1, CDX2, CHD7, CLEC1A, CREB3L3, CTGF, FOXP1, GATA4, GDNF, GMNN, GPM6A, IFI16, MT1, NANOG, PHYHIP, PIK3C2G, PTGER4, RHOX6, RHOX9, RNF24, SALL3, SETDB1, SLC5A9, SNAI1, SNAI2, SOX2, SOX4, SOX17, TBXA2R, TEAD1, TGFBR1, WNK1 | 12 | 12 | Gene Expression, Embryonic Development, Organismal Development |
| 15 | AHR, ANKRD40, C16orf89, CDKN2B, CELA1, CHRNB1, COL4A1, COL4A2, COL6A3, COLQ, CSHL1, DAG1, DCN, DOK7, ESRRG, FBXO32, FMO2, FZD9, GHRH, HMX2, HSD3B4, IFI44, IFNLR1, KDM2B, LIF, MUSK, NSG2, OASL2, RAPSN, SECCTM1B, SIRT1, SLC16A5, SNTA1, TAL1, TRIM63 | 12 | 12 | Developmental Disorder, Hereditary Disorder, Immunological Disease |
| 16 | AP1, AQP2, AQP3, AQP4, ATP8A2, AVPR2, BDH1, CD300LG, CEBPD, DBP, EGF, FOS, HLF, IL15RA, ITGB3, MET, MSX2, NFAT5, NKX2-3, NKX3-1, NOS1, NOS3, PLK2, PODXL, PRDM1, RUNX1, RXRA, SCNN1A, SCTR, SLC6A12, TEF, TLR4, TREM1, UGCG, YWHAZ | 12 | 12 | Organismal Injury and Abnormalities, Renal and Urological Disease, Renal and Urological System Development and Function |
| 17 | APOC3, ARNTL, CACNA1B, CACNB1, CACNB3, CACNB4, CPE, CPNE4, CYP19A1, DLG4, FFAR4, GABRA2, GCGR, GRPR, HTR1A, INS1, INSRR, KCNJ10, LDL-cholesterol, LIPC, LIPG, LPL, LRP5, MOGAT2, NEFL, OPRM1, PRKCI, SCP2, SLC25A5, SLC6A2, SYN1, SYN2, SYN3, TRIB1, VLDL-cholesterol | 10 | 11 | Behavior, Protein Synthesis, Psychological Disorders |


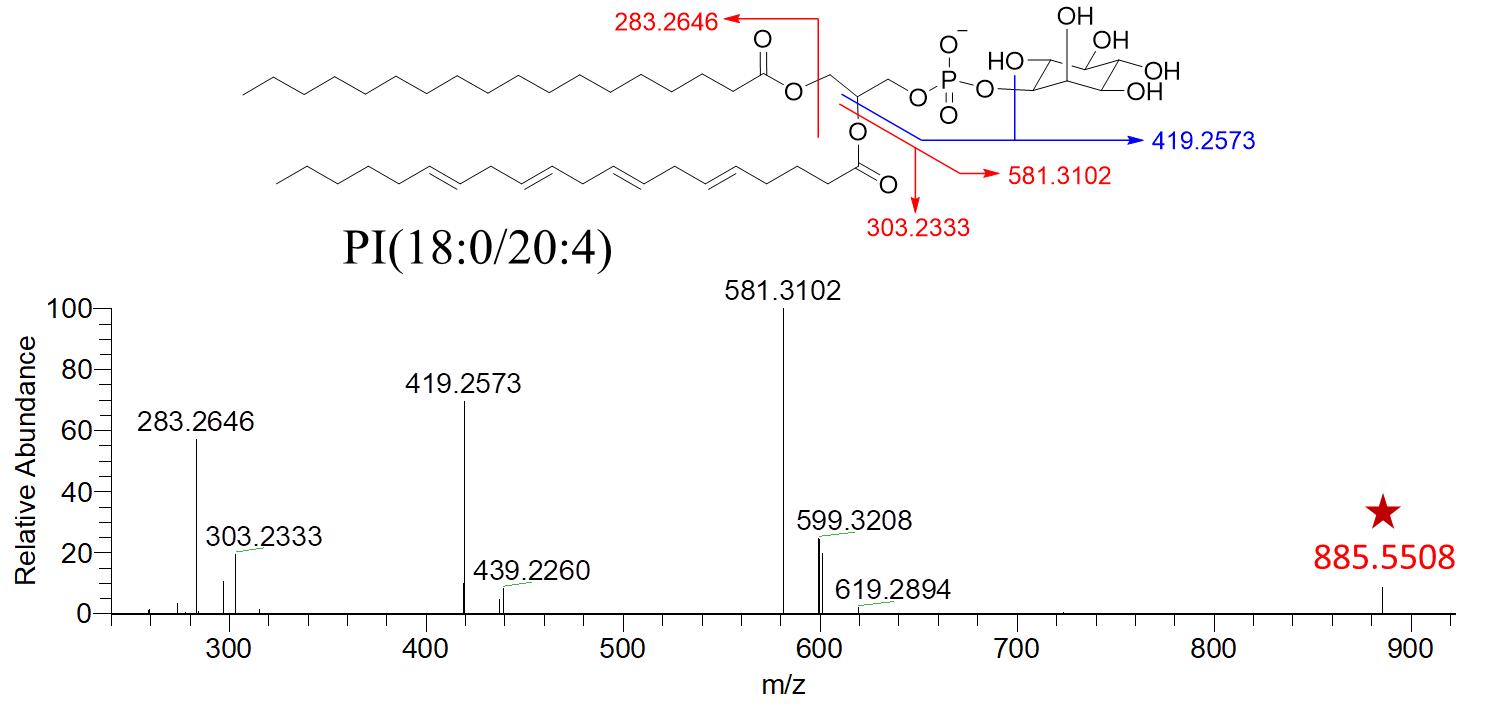


**Figure S1a.** Collision-induced dissociation (CID) at *m/z* 885.5508 attributes to the species as PI(18:0/20:4). Inset shows the fragmentation profile of the species, which also matches with the standard in the lipid database (Ref. 1; [www.lipidmaps.org/](http://www.lipidmaps.org/)).


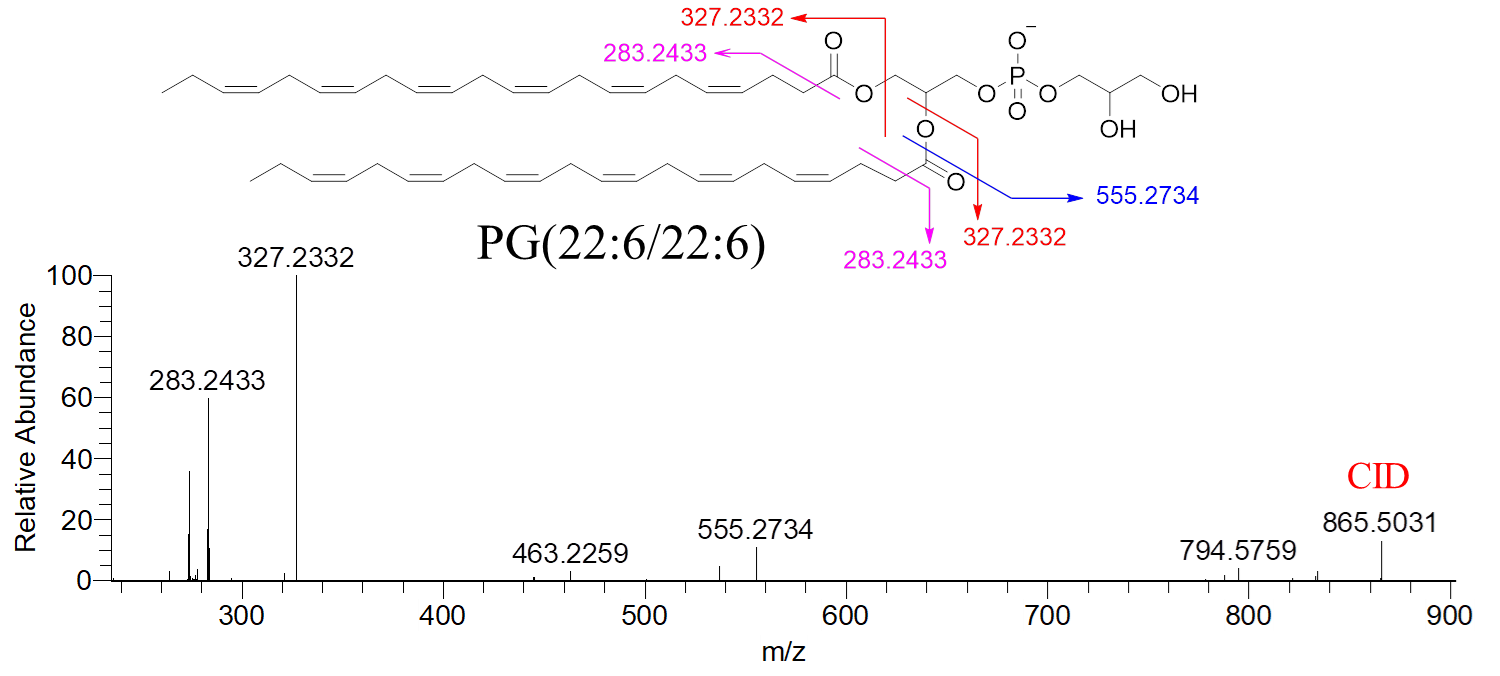


**Figure S1b.** Collision-induced dissociation (CID) at *m/z* 865.5038 attributes to the species as PG(22:6/22:6). Inset shows the fragmentation profile of the species, which also matches with the standard in the lipid database (Ref. 1; [www.lipidmaps.org/](http://www.lipidmaps.org/)).


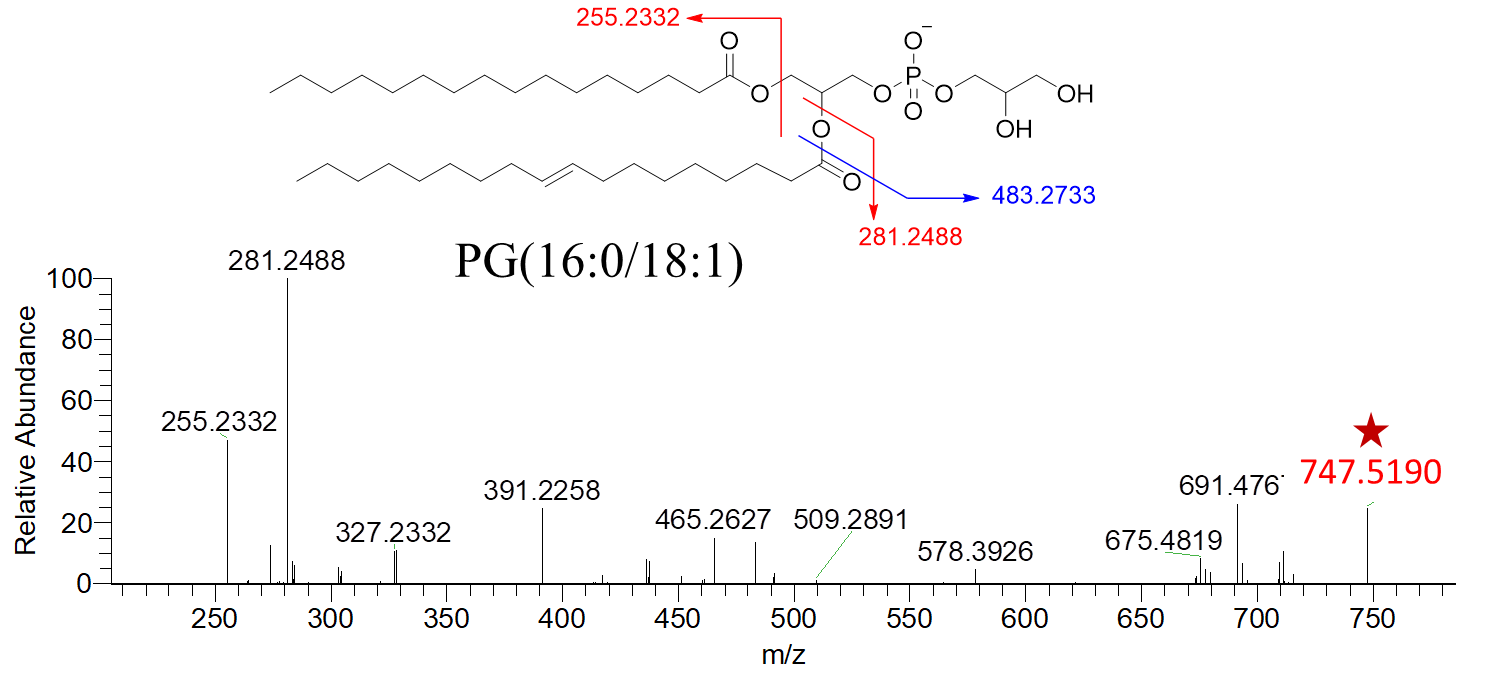


**Figure S1c.** CID at *m/z* 747.5190 attributes to the species as PG(16:0/18:1). Inset shows the fragmentation profile of the species, which also matches with the standard in the lipid database (Ref. 1; [www.lipidmaps.org/](http://www.lipidmaps.org/)).


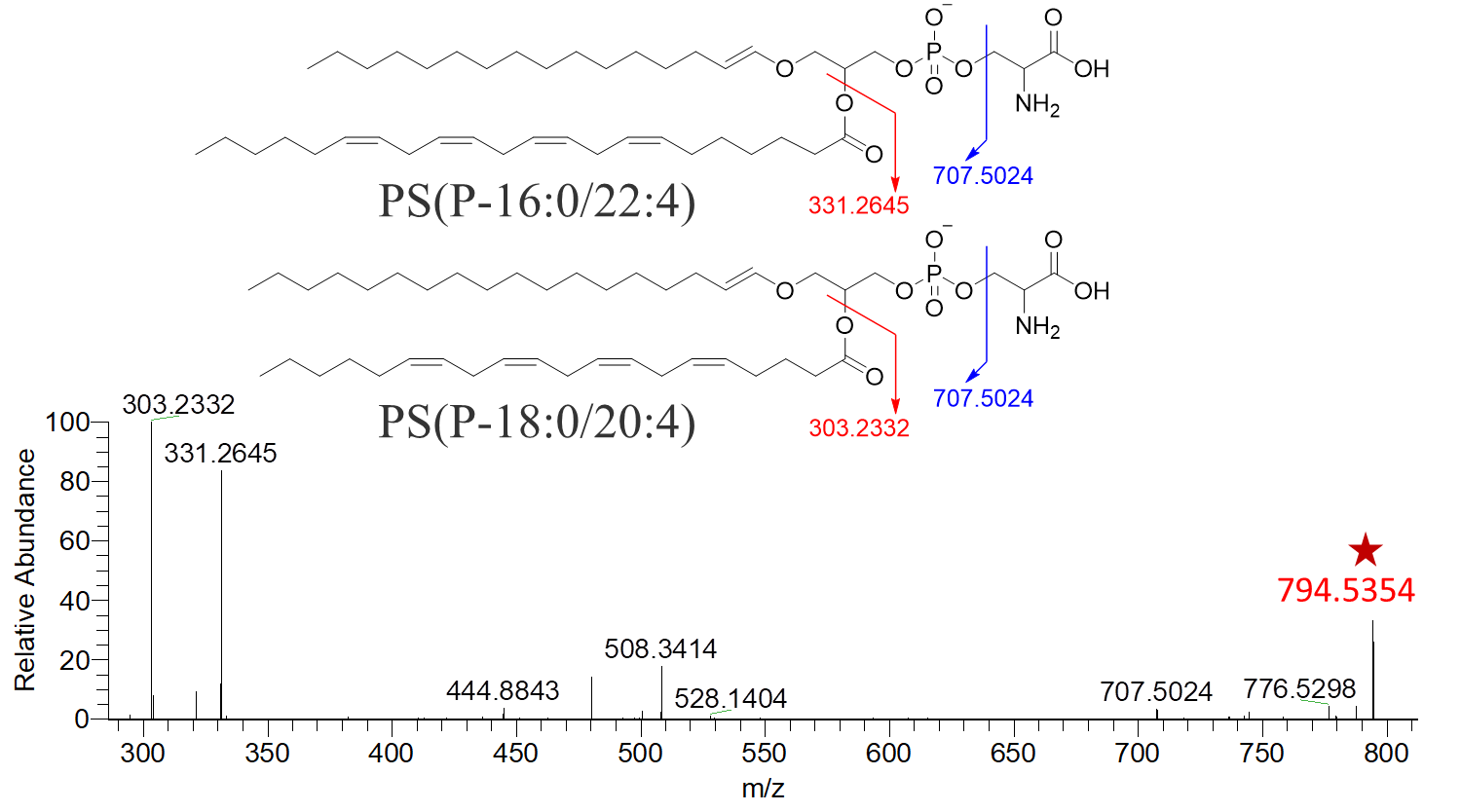


**Figure S1d.** CID at *m/z* 794.5354 attributes to the species as a mixture of PS(P-16:0/22:4) and PS(P-18:0/20:4). Inset shows the fragmentation profile of the species, which also matches with the standard in the lipid database (Ref. 1; [www.lipidmaps.org/](http://www.lipidmaps.org/)).


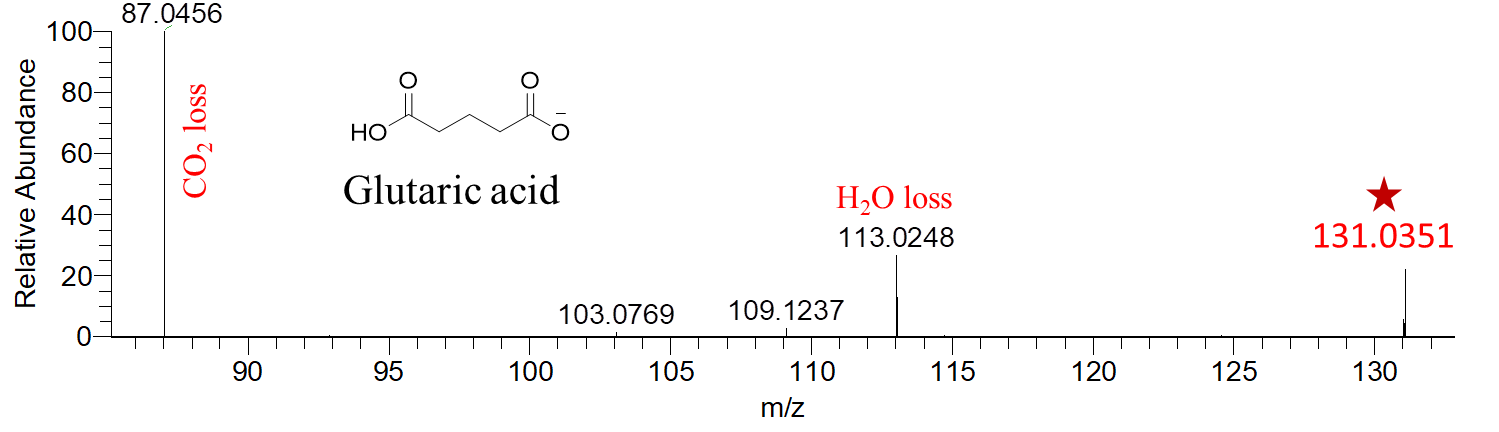


**Figure S1e.** CID at *m/z* 131.0351 attributes to the species as glutaric acid. Inset shows the fragmentation profile of the species, which also matches with the standard in the lipid database (Ref. 1; [www.lipidmaps.org/](http://www.lipidmaps.org/)).


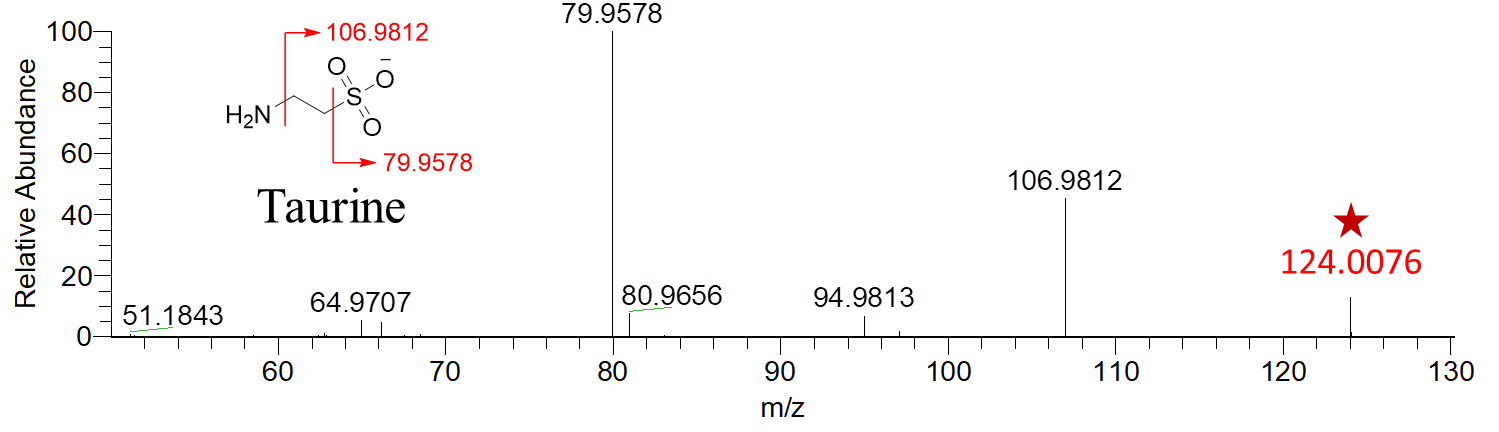


**Figure S1f.** CID at *m/z* 124.0076 attributes to the species as taurine. Inset shows the fragmentation profile of the species, which also matches with the standard in the lipid database (Ref. 1; [www.lipidmaps.org/](http://www.lipidmaps.org/)).


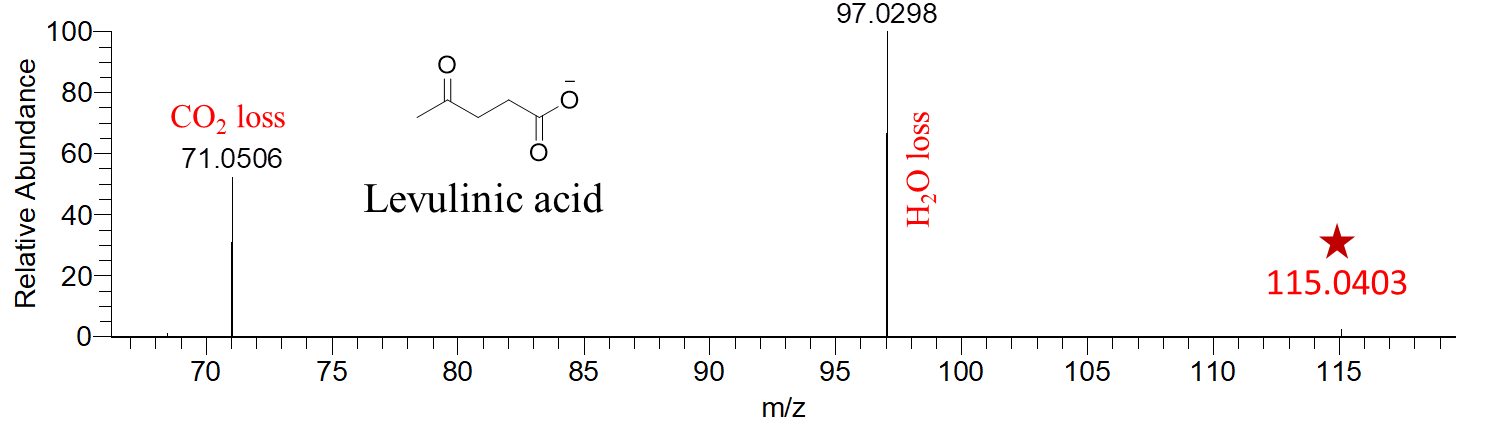


**Figure S1g.** CID at *m/z* 115.0403 attributes to the species as levulinic acid. Inset shows the fragmentation profile of the species, which also matches with the standard in the lipid database (Ref. 1; [www.lipidmaps.org/](http://www.lipidmaps.org/)).


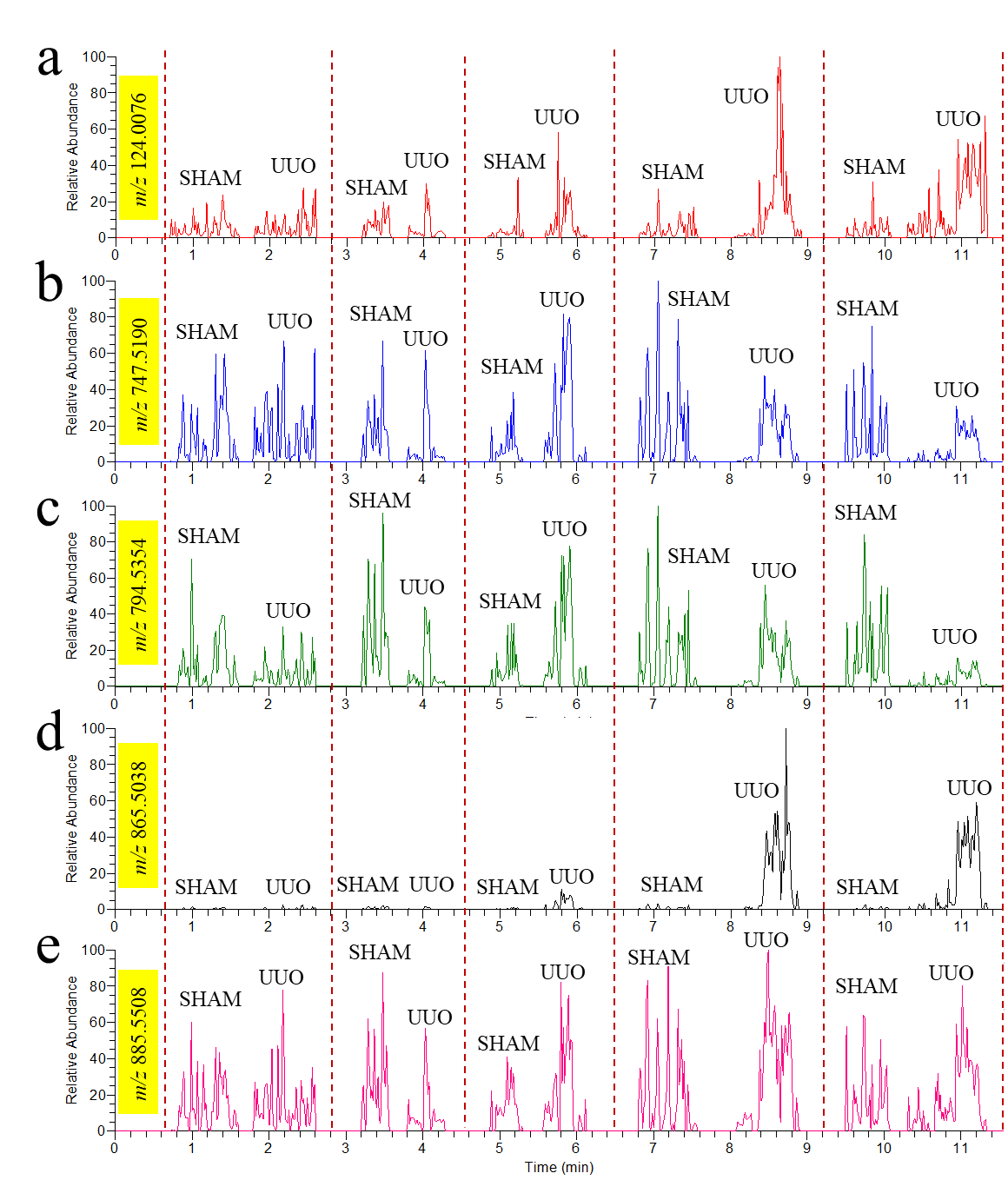


**Figure S2.** Extracted ion chronograms of (a) taurine, (b) PG(16:0/18:1), (c) PS(P-16:0/22:4), PS (P-18:0/20:4), (d) PG(22:6/22:6), and (e) PI(18:0/20:4) over sequential scribble scanning by DESI-MS of representative five pairs (sham control and UUO) of kidney tissue specimens at five different time points.


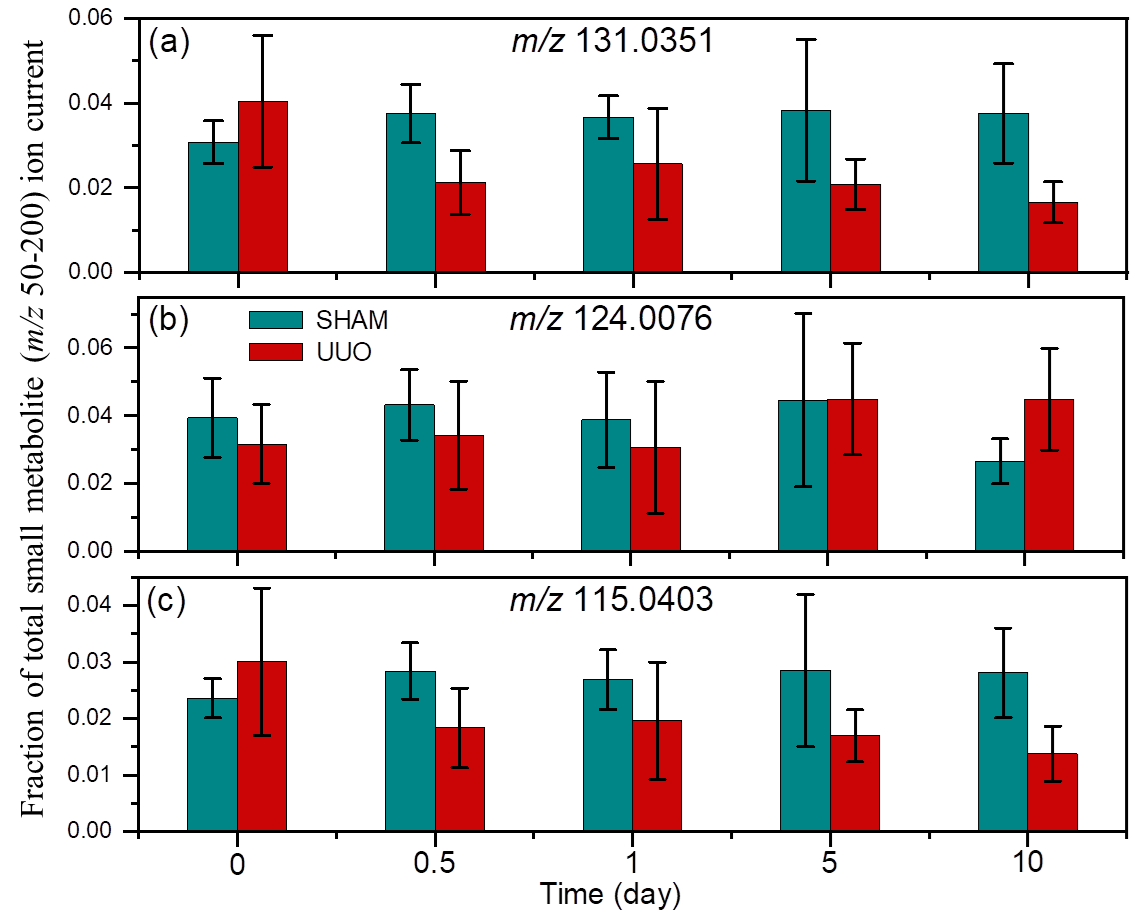


**Figure S3.** Time-dependent (0-10 d) abundance (normalized with total small metabolite ion current; Figure 1) of (a) glutaric acid (*m/z* 131.0351), (b) taurine (*m/z* 124.0076), (c) levulinic acid (*m/z* 115.0403) in UUO (red bar) and sham control (green bar). The data at each time point of each panel represent the mean ± SD calculated from eight mice.


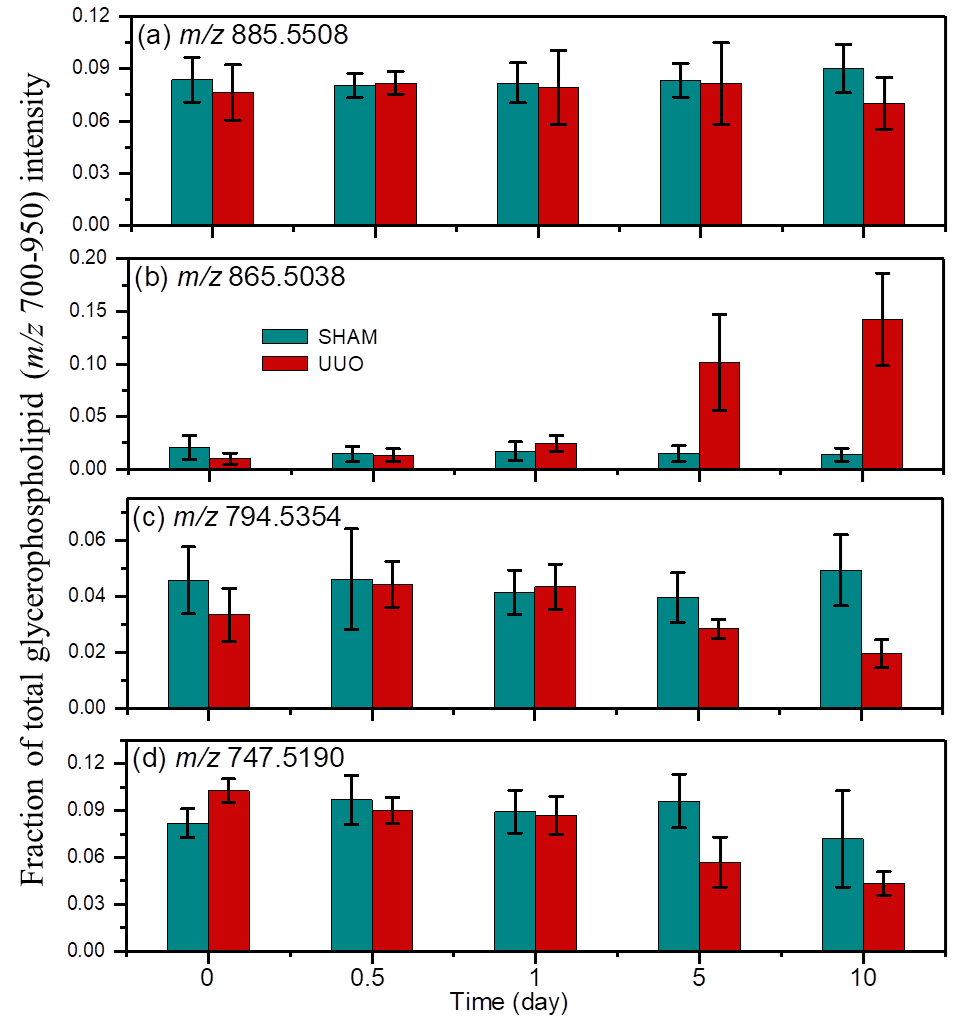


**Figure S4.** Time-dependent (0-10 d) abundance (normalized with total glycerophospholipid ion current; Figure 1) of (a) PI(18:0/20:4) [*m/z* 885.5508], (b) PG(22:6/22:6) [*m/z* 865.5038], (c) PS(P-16:0/22:4)/(P-18:0/20:4) [*m/z* 794.5354], and (d) PG(16:0/18:1) [*m/z* 747.5190] in UUO (red bar) and sham control (green bar). The data at each time point of each panel represent the mean ± SD calculated from eight mice.

**
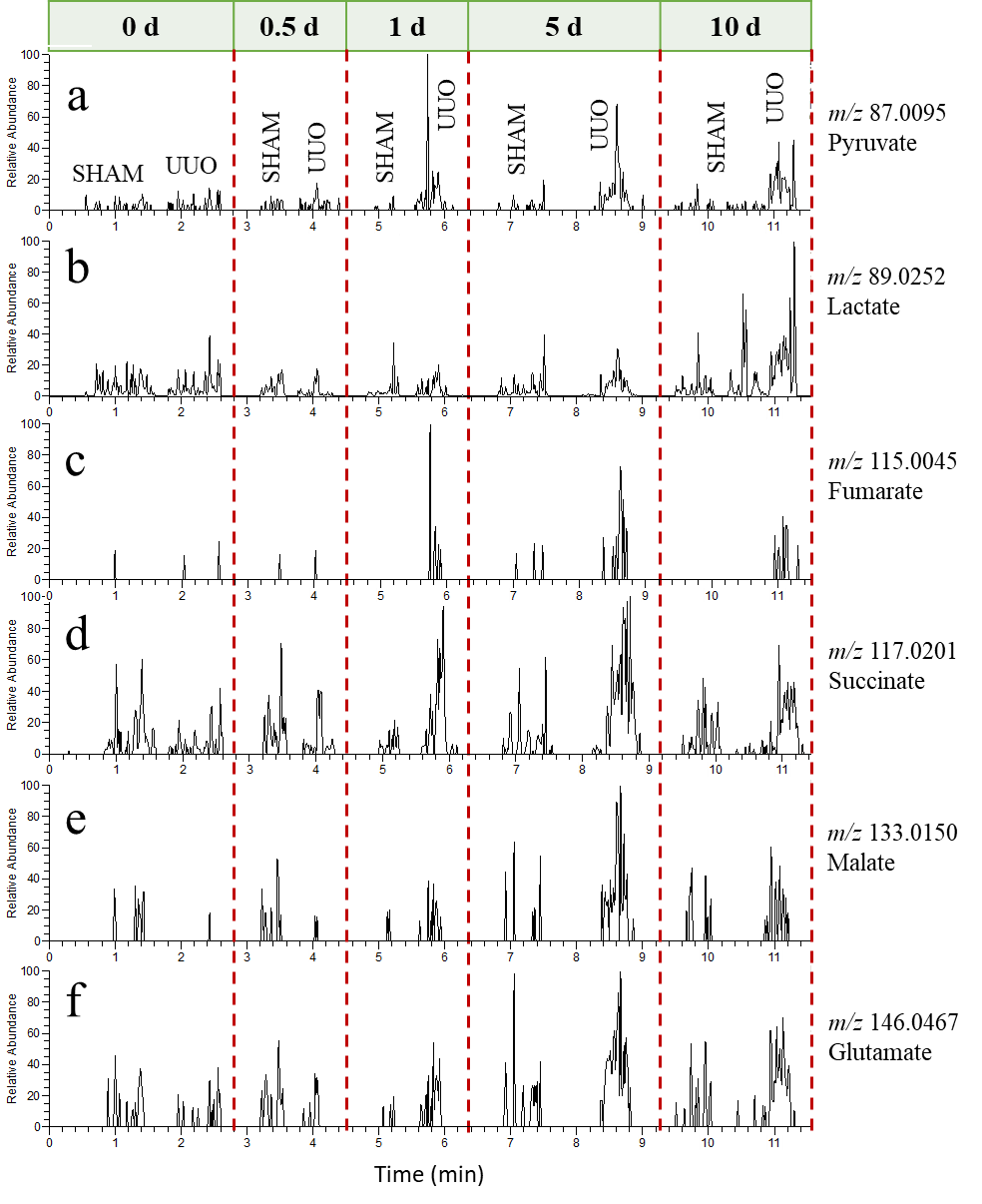
Figure S5.** Extracted ion chronograms of (a) pyruvate, (b) lactate, (c) fumarate, (d) succinate, (e) malate, and (f) glutamate over sequential scribble scanning by DESI-MS of representative five pairs (sham control and UUO) of kidney tissue specimens at five different time points. The observed *m/z* value of an individual molecular species is given on the right side of the corresponding panel.

**Reference**

1. Fahy E., *et al*: Update of the LIPID MAPS comprehensive classification system for lipids. *Journal of Lipid Research* **50,** S9-S14, DOI: 10.1194/jlr.R800095-JLR200, (2009).
